# Supplementary material for: Multiple myeloma associated long non-coding RNA PLUM confers chemoresistance by enhancing PRC2 mediated UPR pathway activation
Source: Nat Commun. 2025 Sep 1;16:8155. doi: 10.1038/s41467-025-63256-x (PMC12402260; doi:10.1038/s41467-025-63256-x)
Supplement: Supplementary file 1 — Supplementary Information [file 41467_2025_63256_MOESM1_ESM.docx]

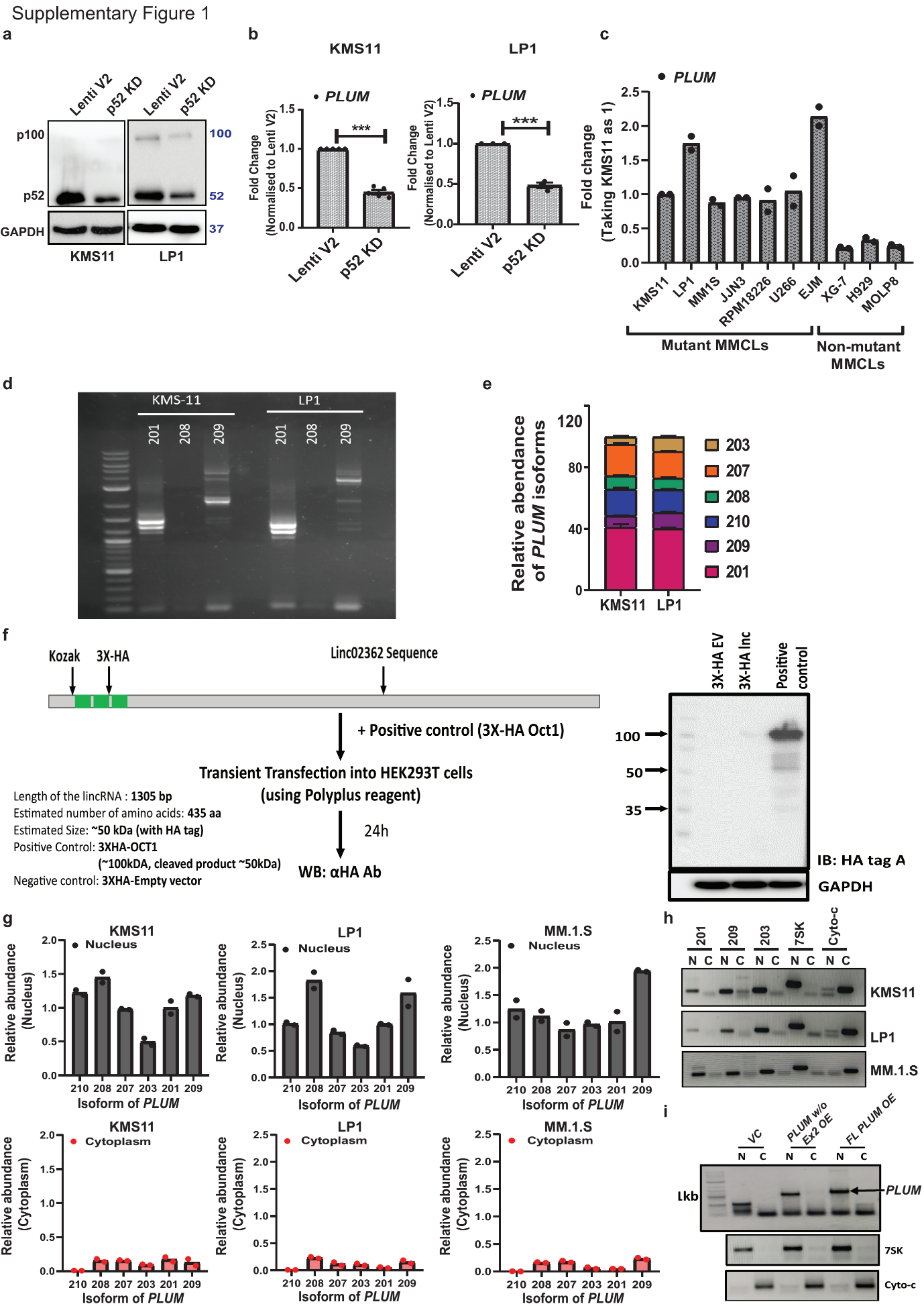
 **Supplementary Figure 1: Identification and characterization of *PLUM* in MMCLs. (a).** Level of NF-ĸB/p52 protein in empty vector (Lenti-V2) and Lenti-V2 CRISPR-p52 knock down MMCLs (KMS11 and LP1) (N=5 for KMS11 and N=3 for LP1). (b). Densitometric analysis (mean fold change ±SEM) for NF-ĸB/p52 band in western blots of supp figure 1a. Analysis done using GelQuant.NET. The values for p52 band were normalised to GAPDH band (N=5 for KMS11 and N=3 for LP1; two-sided unpaired student’s t test; p-values – KMS11: 0.0001, LP1: 0.0001). (**c).** Endogenous expression level of *PLUM* in NF-ĸB+ (KMS11, LP1, MM.1.S, JJN3, RPMI8226, U266, EJM) and NF-ĸB- (XG-7, H929, MOLP8) MMCLs (N=2 biological replicates). **(d).** Gel image showing the bands for 5′/3′ RACE PCR products for *PLUM* which matched with isoform 201, 208 and 209 from Ensembl database (upon sequencing) in two MMCLs (KMS11 and LP1). **(e).** Relative abundance of different isoforms of *PLUM* in MMCLs (KMS11 and LP1) (N=2 biological replicates). (**f).** Graphical representation of the experimental design to study the coding potential of *PLUM*. Expression level of HA-*PLUM* and HA-Oct4 (positive control) protein probed with HA antibody showing no expression of *PLUM* protein or *PLUM* peptides. **(g).** Relative abundance of different isoforms of *PLUM* in the nuclear fraction (Top: Black bars) normalised to 7SK transcripts and in cytoplasmic fraction (Bottom: Red bars) normalised to cyto-c transcripts of three MMCLs (KMS11, LP1 and MM.1.S) (N=2 biological replicates). **(h).** Expression level of different isoforms of *PLUM* with control RNAs (7SK and cyto-c) in nuclear and cytosolic fractions of three MMCLs (KMS11, LP1 and MM.1.S) (N=2 biological replicates). **(i).** Expression level of two exogenously overexpressed major isoforms of *PLUM* in nuclear and cytosolic fractions compared to VC transduced cells (N=2 biological replicates).


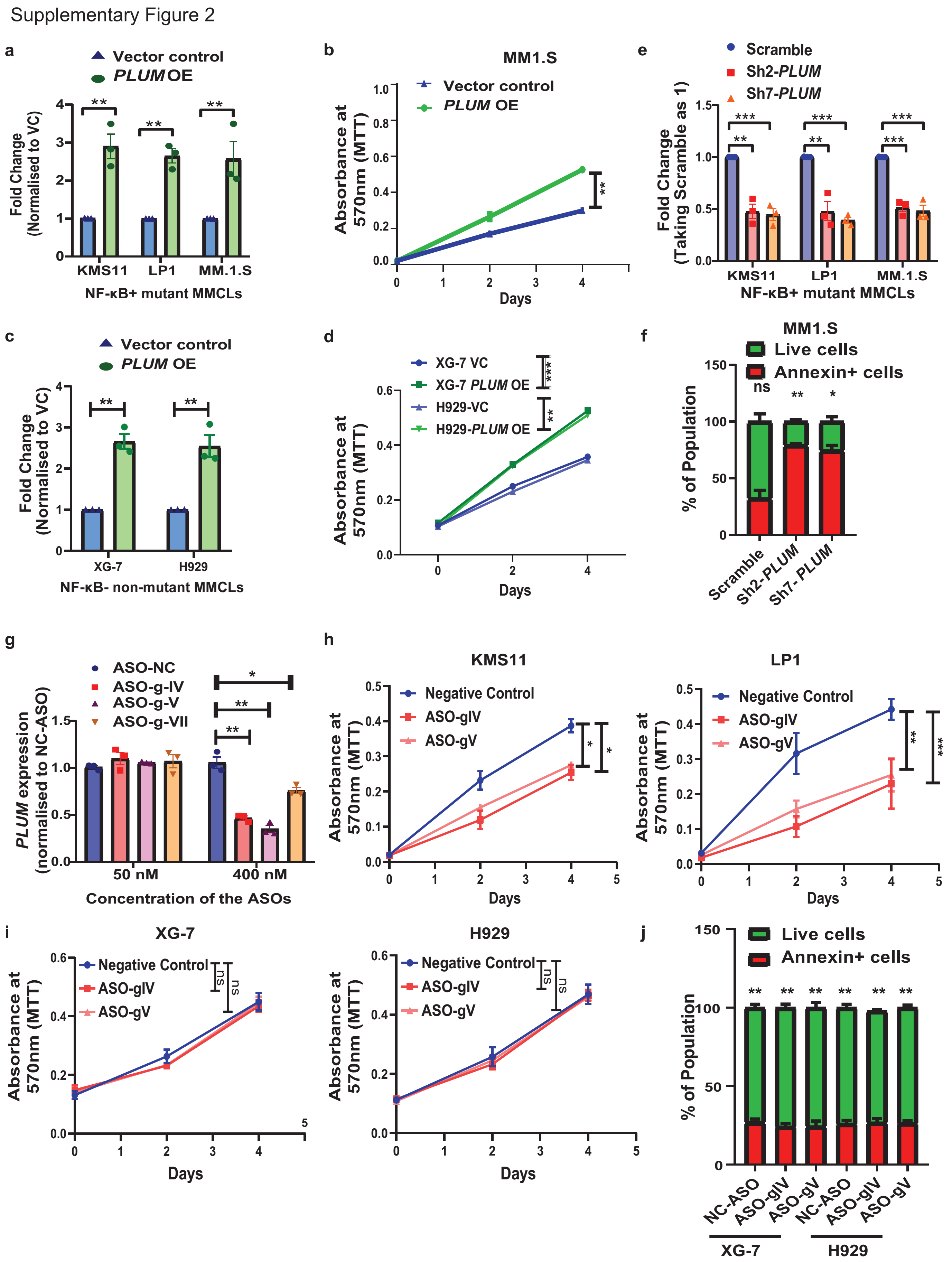
 **Supplementary Figure 2: Functional characterization of *PLUM* in MMCLs. (a).** Fold change enrichment (mean ± SEM) of FL*-PLUM* in overexpressed NF-κB+ MMCLs (KMS11, LP1 and MM.1.S) compared vector control (VC) (N=3, two-sided unpaired student’s t test; p-values – KMS11: 0.005, LP1: 0.005, MM.1.S: 0.005). **(b).** Proliferation rate (mean ± SEM) of MM.1.S cells overexpressed with FL-*PLUM* relative to VC (N=3, two-way ANOVA; p-values – MM.1.S: 0.0012). (**c).** Fold change enrichment of FL*-PLUM* (mean ± SEM) in overexpressed NF-κB- MMCLs (XG-7 and H929) compared VC (N=3, two-sided unpaired student’s t test; p-values – XG-7: 0.0008, H929: 0.0045). **(d).** Proliferation rate (mean ± SEM) of NF-κB- MMCLs (Xg-7 and H929) overexpressed with FL *PLUM* relative to VC till day 4 (N=3, two-way ANOVA; p-values – XG7: 0.0001, H929: 0.0019). **(e).** Expression level (mean ± SEM) of FL *PLUM* in sh-scramble, sh2-*PLUM* and sh7-*PLUM* KD NF-κB+ MMCLs (KMS11, LP1 and MM.1.S) (N=3, two-sided unpaired student’s t test; p-values – KMS11: scramble sh2-*PLUM*: 0.0016, scramble versus sh7-*PLUM*: 0.0016; LP1: scramble versus sh2-PLUM: 0.0046, scramble versus sh7-*PLUM*: 0.0001; MM.1.S: scramble sh2-*PLUM*: 0.0003, scramble versus sh7-*PLUM*: 0.0007). (**f).** Percentage of live and apoptotic cells (Annexin V+) (mean ± SEM) in sh-scramble versus sh-*PLUM* MM.1.S cells at day 6 post transduction (N=3, two-sided multiple t-test; p-values – MM.1.S scramble: 0.003, sh2: 0.001, sh7: 0.001). (**g).** Relative expression levels of *PLUM* (mean ± SEM) post treatment with degradative ASOs at concentrations of 50nM and 400nM. (N=3, two-sided unpaired student’s t test; p-values – At 400nM, ASO-NC versus ASO-gIV: 00007, ASO-NC versus ASO-gV: 0.0006). (**h).** Proliferation rate (mean ± SEM) of NF-κB+ MMCLs (KMS11 and LP1) treated with NC-ASO and degradative ASOs (ASO-gIV and ASO-gV) for 36 h (N=3, two-way ANOVA; p-values – For KMS11, NC-ASO versus ASO-gIV: 0.04, NC-ASO versus ASO-gV: 0.013; For LP1, NC-ASO versus ASO-gIV: 0.0004, NC-ASO versus ASO-gV: 0.001).(**i).** Proliferation rate (mean ± SEM) of XG-7 and H929 cells treated with NC-ASO and degradative ASO-gIV/ ASO-gV (N=3, two-way ANOVA; p-values - non-significant). (**j).** Percentage of live and apoptotic cells (AnnexinV+) (mean ± SEM) in NC-ASO and degradative ASO-gIV, g-V treated NF-κB - MMCLs (XG-7 and H9292) (N=3, two-sided multiple t-test; p-values with biological replicates 0.001).


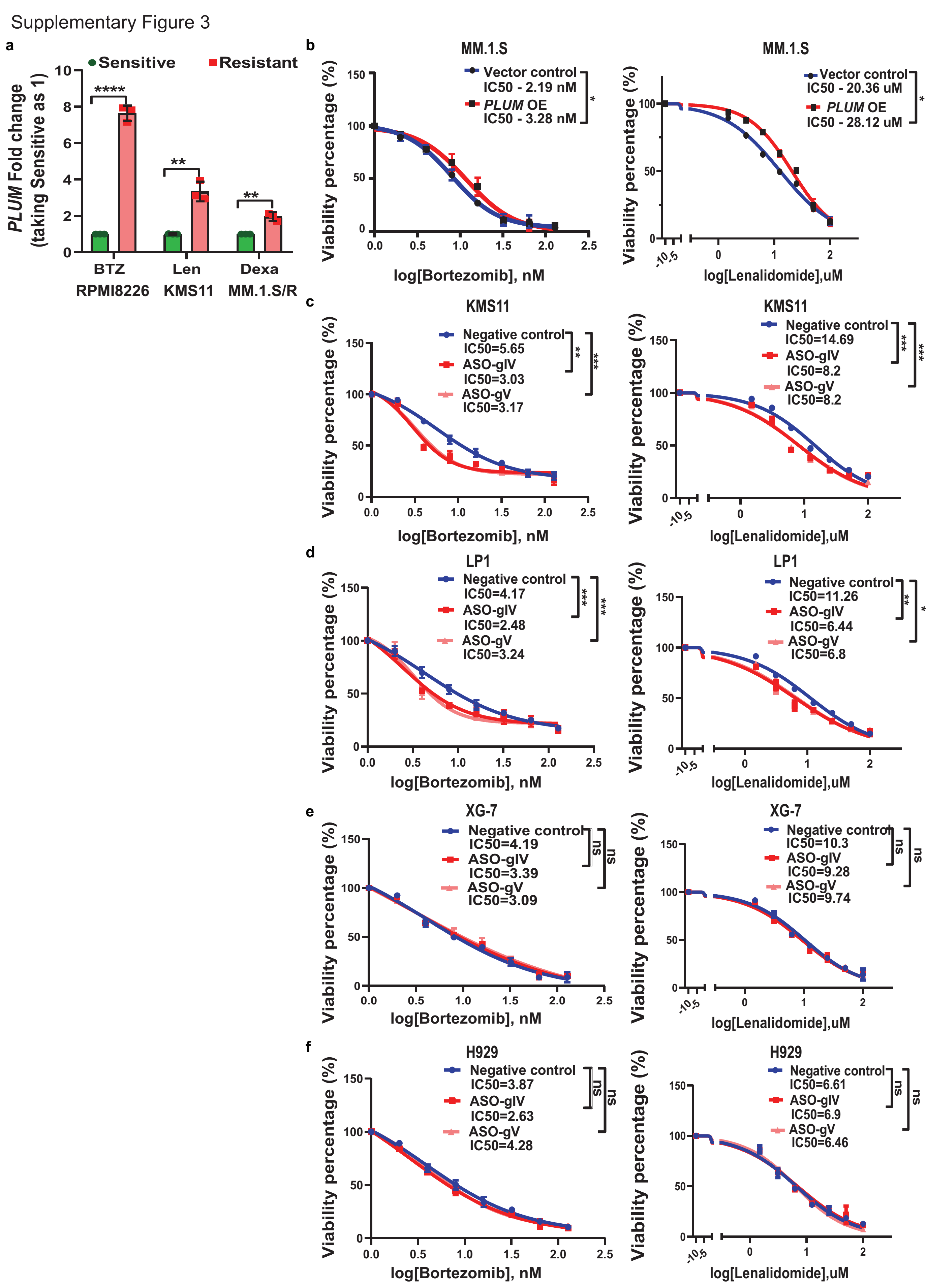
 **Supplementary Figure 3: *PLUM* expression is correlated with chemoresistance in MMCLs**. (**a).** Expression level (mean ± SEM) of *PLUM* in BTZ-R (RPMI8226), Len-R (KMS11) and Dexa-R (MM.1R) versus its parental sensitive cells (N=3, two-sided unpaired student’s t test; p-values – BTZ-R versus BTZ-S: 0.0001, Len-R versus Len-S: 0.0017, Dexa-R versus Dexa-S: 0.0025). (**b).** Drug sensitivity IC_50_ survival curve (mean ± SEM) for *PLUM* overexpressed MM.1.S cell line compared to vector control in response to BTZ treatment for 24h and Len treatment for 4 days (N=3, two-way ANOVA; p-values – BTZ: 0.045 and Len: 0.043). (**c,d).** Drug sensitivity IC_50_ survival curve (mean ± SEM) for NC-ASO and degradative ASO (ASO-gIV and ASO-gV) treated cells in response to BTZ treatment for 24h and Len treatment for 4 days in KMS11 and LP1 cells respectively (N=3, two-way ANOVA; p-values – For KMS11 BTZ treatment, NC-ASO versus ASO-gIV: 0.005, NC-ASO versus ASO-gV: 0.0005; For KMS11 Len treatment, NC-ASO versus ASO-gIV: 0.0005, NC-ASO versus ASO-gV: 0.0005; For LP1 BTZ treatment, NC-ASO versus ASO-gIV: 0.0005, NC-ASO versus ASO-gV: 0.0005; For LP1 Len treatment, NC-ASO versus ASO-gIV: 0.005, NC-ASO versus ASO-gV: 0.045). (**e,f):** Drug sensitivity IC_50_ survival curve (mean ± SEM) for NC-ASO and degradative ASO (ASO-gIV and ASO-gV) treated cells in response to BTZ treatment for 24h and Len treatment for 4 days in XG-7 and H929 cells respectively (N=3, two-way ANOVA; p-values - ns: non-significant).


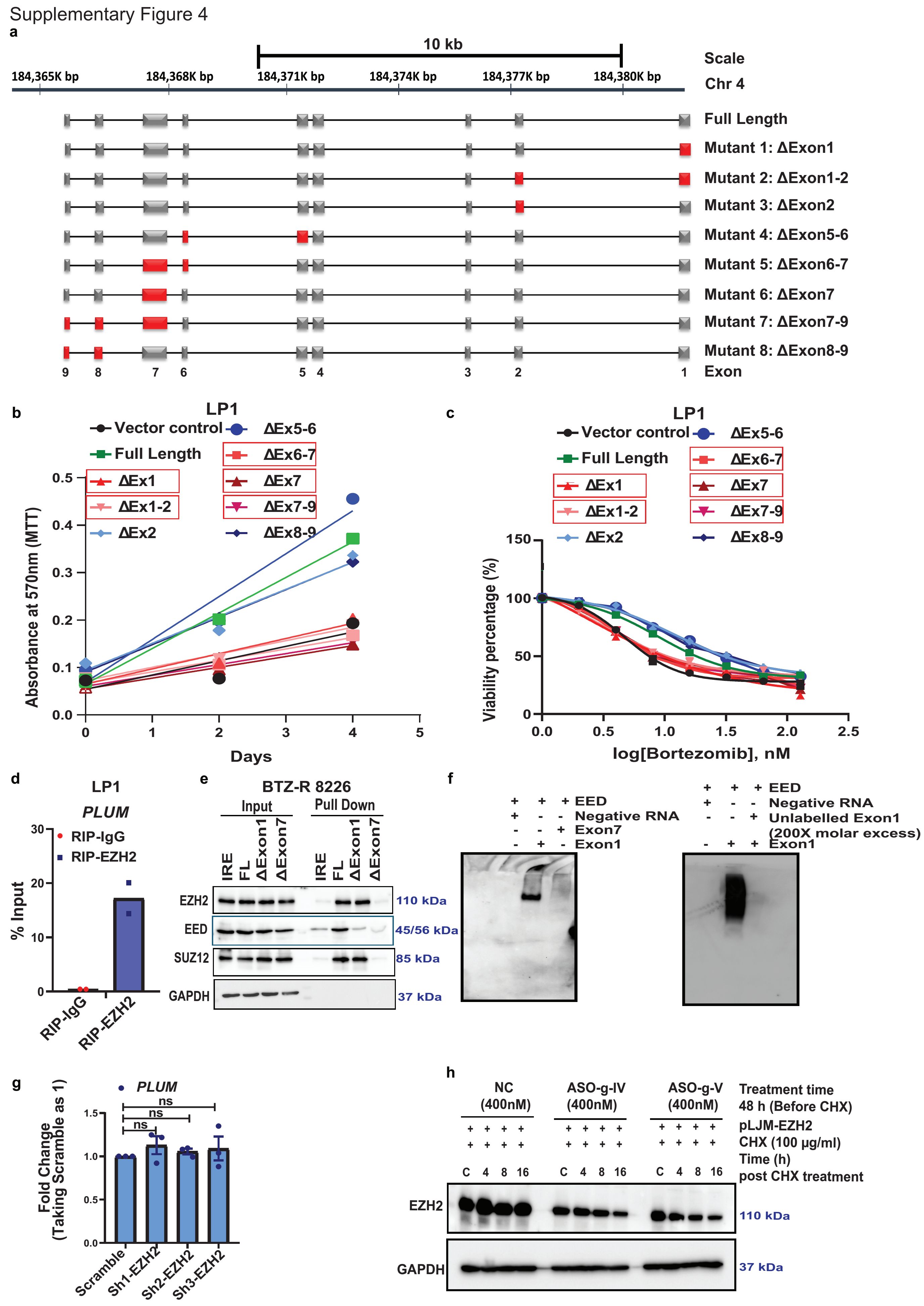
 **Supplementary figure 4: Mutagenesis design for *PLUM* expression construct and characterization of the activity of ASOs in MM cells*.* (a):** Line diagram representing different exon deletion mutants of *PLUM* (Boxes: exons; Lines: introns; red boxes: deleted exons), generated by site-directed mutagenesis. **(b).** Proliferative rate of 8 exon deletion mutants of *PLUM* overexpressed LP1 cells compared to FL *PLUM* and vector control (VC). Red box: *PLUM* mutants with no proliferative phenotype compared to VC (N=2 biological replicates). **(c).** Drug sensitivity IC_50_ survival curve of 8 exon deletion mutants of *PLUM* overexpressed LP1 cells compared to FL-*PLUM* and VC post BTZ treatment (24h). Red box: *PLUM* mutants sensitive to BTZ treatment compared to VC (N=2 biological replicates). (**d).** RIP-qPCR validation of *PLUM* with EZH2 and IgG antibody in LP1 cells (N=2 biological replicates). **(e).** Immunoblots showing differential binding of PRC2 complex proteins (EZH2, EED and SUZ12) with FL *PLUM*, ΔExon1 and ΔExon7 mutants of *PLUM* using BTZ-R 8226 nuclear lysates. IRE transcript: negative control; GAPDH: input protein loading control (N=2 biological replicates). (**f).** RNA-EMSA blot of purified WT-EED protein with biotinylated exon1 and exon7 region of *PLUM* RNA. Labelled negative control (NC) RNA was used as control. RNA-EMSA blot of purified WT-EED protein with biotinylated exon1 region and unlabelled exon1 region (200X molar concentration) of *PLUM* RNA. (**g).** Expression level (mean fold change ± SEM) of *PLUM* in sh1-*EZH2*, sh2-*EZH2* and sh3-*EZH2* KD cells compared to sh-scramble taken as 1 (N=3, two-sided unpaired student’s t test; ns: non-significant). (**h).** Level of exogenously overexpressed EZH2 (pLJM-EZH2) protein in NC-ASO and degradative ASO (g-IV and g-V) treated NF-κB+ KMS11 cells at different time points of treatment with cycloheximide (100µg/ml) (N=2 biological replicates).
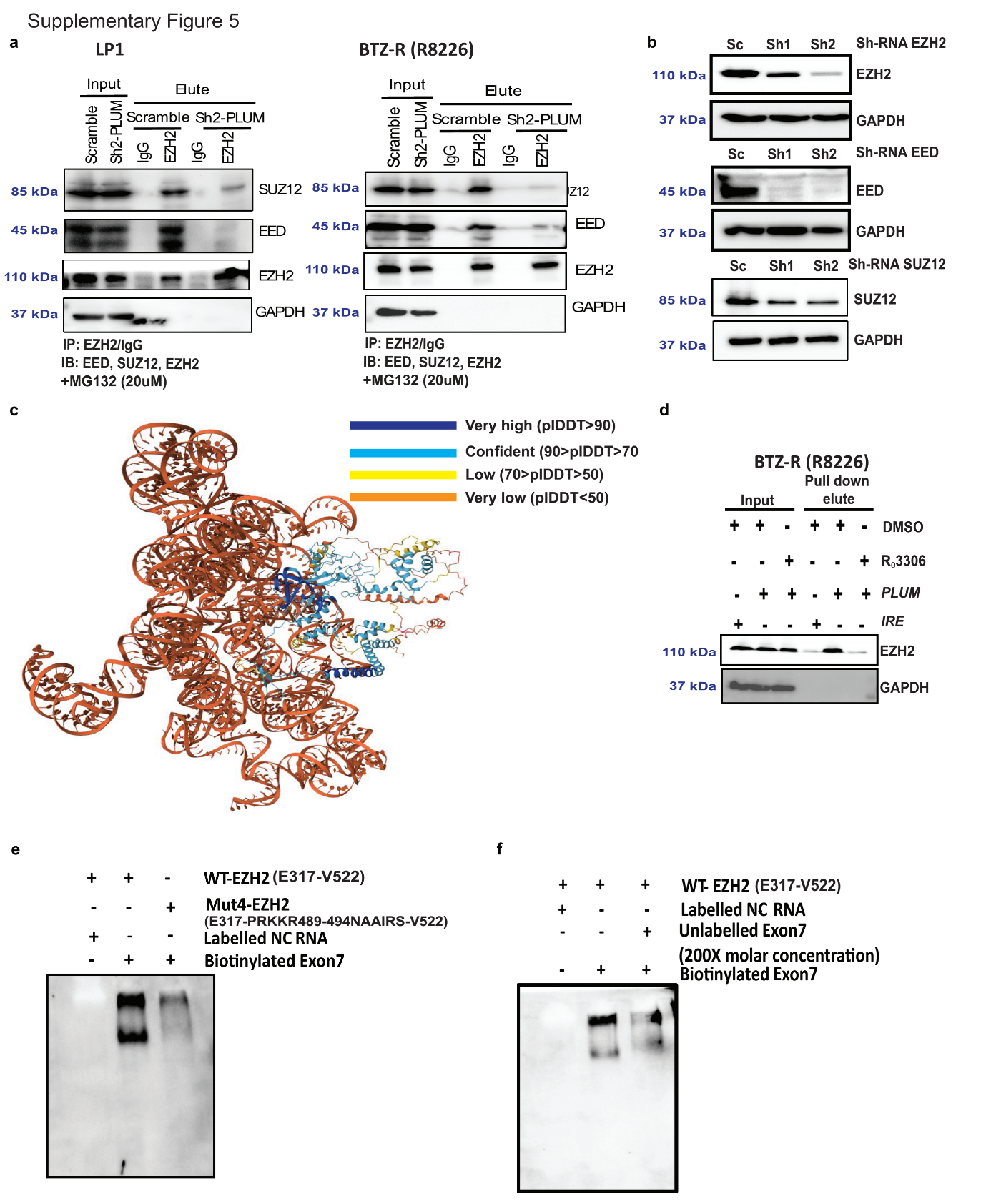
**Supplementary Figure 5: *PLUM* facilitates formation of PRC2 complex via direct interaction with the disordered region of EZH2.** (**a).** Levels of EED and SUZ12 proteins co-immunoprecipitated (Co-IP) by EZH2 antibody in sh-scramble and sh2- *PLUM* KD LP1 and BTZ-R 8226 cell lines post day 2 after selection. (+MG132-20µM); IgG: IP control, GAPDH: input protein loading control (N=2 biological replicates). (**b).** Expression level of EZH2, EED and SUZ12 proteins in sh-scramble and sh-EZH2/EED/SUZ12 transduced KMS11 cells respectively. sh2-knock down cells were used for RNA-protein pull down assay. (**c).** The docked structure of the *PLUM*-EZH2 complex generated using AlphaFold3. The colour shading of the structure represents the pIDDT confidence score of the structure. The pTM score (predicted TM-score for the full structure) is 0.35 and the ipTM (predicted TM-score of the interfaces between subunits) is 0.17. (**d).** Binding level of EZH2 protein pulled with FL *PLUM* in CDK1 inhibitor (R₀3306) treated BTZ-R 8226 cell line. DMSO: vehicle control, IRE transcript: negative control, GAPDH: input protein loading control (N=2 biological replicates). (**e).** RNA-EMSA blot of purified truncated WT EZH2 (E317-V522) and truncated Mut4-EZH2 (E317-PRKKR489-494NAAIRS-V522) protein with biotinylated exon 7 region of *PLUM* RNA. Labelled negative control (NC) RNA was used as control. (**f).** RNA-EMSA blot of purified truncated WT EZH2 (E317-V522) protein with biotinylated exon7 region of *PLUM* and 200-fold molar excess of unlabelled exon 7 region of *PLUM*.


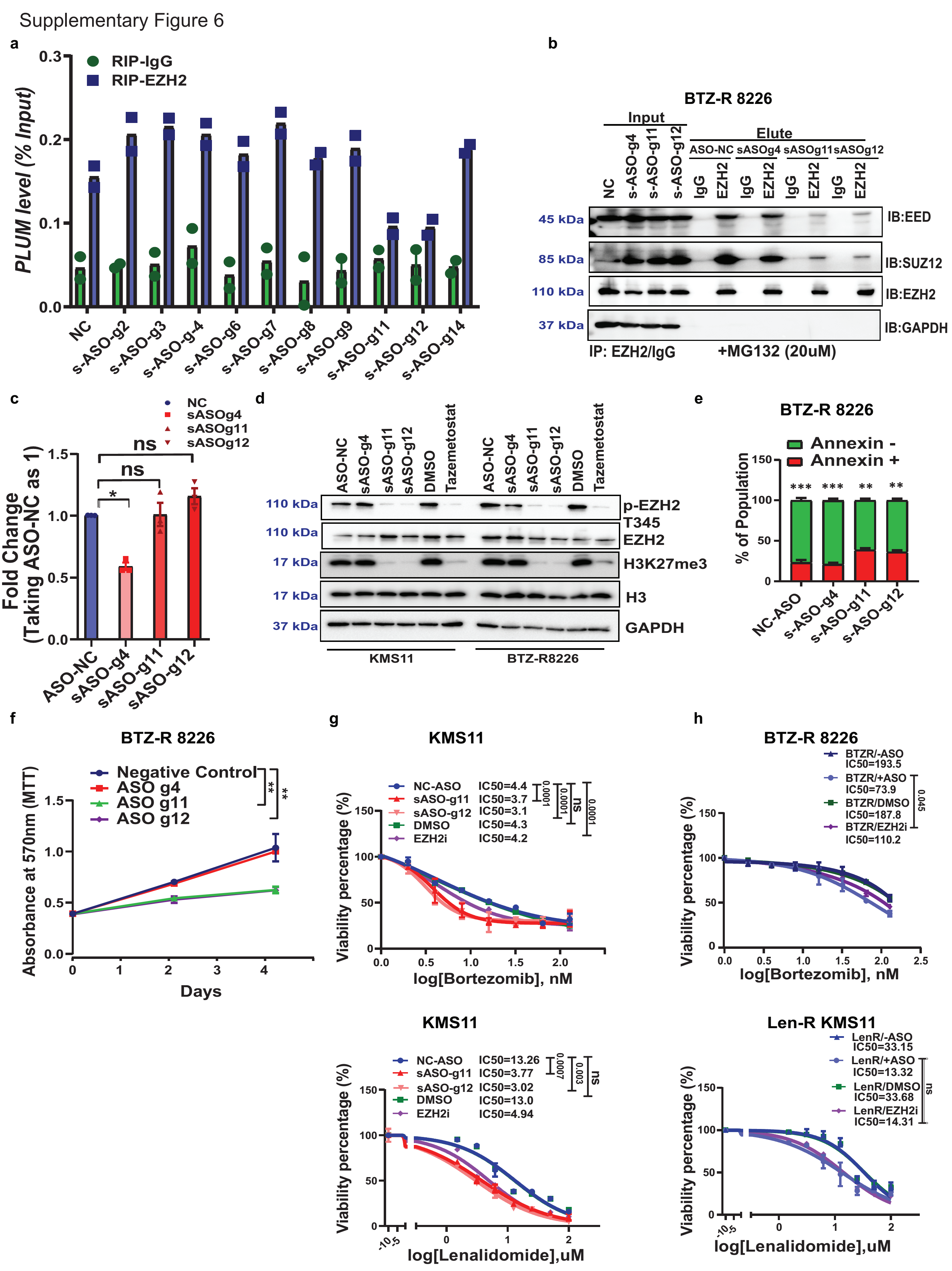
**Supplementary figure 6: Steric ASOs mediated disruption of *PLUM*-EZH2 complex resensitize MMCLs to chemotherapeutic drugs. (a):** RIP-qPCR validation of *PLUM* with EZH2 antibody compared to IgG antibody in KMS11 post treatment with NC-ASO and steric ASOs (s-ASO-g2, s-ASO-g3, s-ASO-g4, s-ASO-g6, s-ASO-g7, s-ASO-g8, s-ASO-g9, s-ASO-g11 and s-ASO-g12) for 3 days (N=2 biological replicates). **(b).** Level of EED and SUZ12 proteins co-immunoprecipitated (Co-IP) by EZH2 antibody in NC-ASO, s-ASO-g4, s-ASO-g11 and s-ASO-g12 treated BTZ-R 8226 cell line. (+MG132-20µM); IgG: IP control, GAPDH: input protein loading control (N=2 biological replicates). **(c).** Expression level (mean ± SEM) of *PLUM* in KMS11 cells treated with NC-ASO, s-ASO-g4, s-ASO-g11 and s-ASO-g12 for 3days. (N=3, two-sided unpaired student’s t test; p-values – ASO-NC versus ASO-g4: 0.032, ns: non-significant). **(d).** Level of p-EZH2/EZH2, H3K27me3/H3 and GAPDH proteins in steric ASOs (0.5 µM) and EZH2 inhibitor (Tazemetostat) (0.5µM) treated cell lines (KMS11 and BTZ-R 8226). ASO-NC and DMSO are used as negative control for s-ASO and EZH2 inhibitor treatment respectively (N=3 biological replicates). **(e).** The percentage (mean ± SEM) of annexin+ cells and annexin– cells post treatment with steric ASOs in BTZ-R 8226 cells (N=4, p values determined by two-sided multiple t-test; p-values - NC-ASO: 0.0001, s-ASO-g4: 0.0001, s-ASO-g11: 0.001, s-ASO-g12: 0.001). **(f).** Proliferation rate (mean ± SEM) of BTZ-R 8226 cells treated with NC-ASO and steric ASOs till day 4 (N=3, two-way ANOVA; p-values - NC Vs s-ASO-g4: non-significant, NC Vs s-ASO-g11: 0.005 and NC Vs s-ASO-g12: 0.005). (**g).** Drug sensitivity IC50 survival curve (mean ± SEM) for steric ASOs and EZH2i (Tazemetostat) treated KMS11 cells in response to BTZ treatment for 24h and Len treatment for 4 days respectively (N=3, two-way ANOVA; p-values marked in the figure). (**h).** Drug sensitivity IC50 survival curve (mean ± SEM) for steric ASOs and EZH2i (Tazemetostat) treated BTZ-R 8226 cells in response to BTZ treatment for 24h and Len-R KMS11 cells in response to Len treatment for 4 days respectively (N=3, two-way ANOVA; p-values - BTZ-R/+ASO-g112 versus BTZ-R/EZH2i: 0.045, LenR/+ASO versus LenR/EZH2i: non-significant).


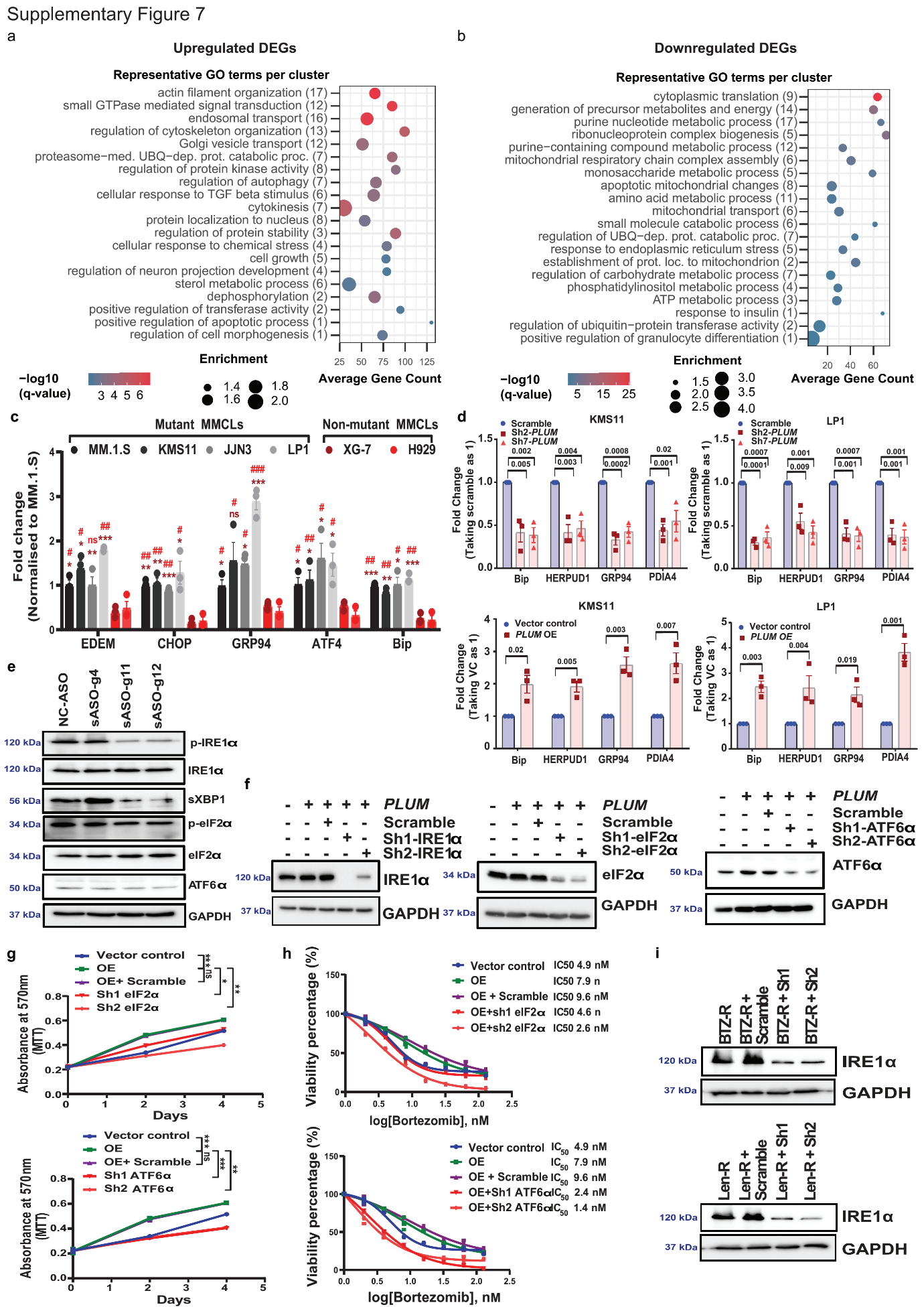
**Supplementary Figure 7: *PLUM* expression level regulates the activation of UPR pathway*.* (a).** Enriched biological processes among upregulated DEGs upon *PLUM* knockdown in KMS11 cell line. (**b).** Enriched biological processes among downregulated DEGs upon *PLUM* knockdown in KMS11 cell line. **(c).** Expression level (mean ± SEM) of UPR target genes (*EDEM, CHOP, GRP94, ATF4 and Bip*) in NF-ĸB+ and NF-ĸB- MMCLs. Fold changes have been normalised to MM.1.S sample (N=3, two-sided unpaired student’s t test; *****: p value for mutant MMCLs versus XG-7, **#**: p value for mutant MMCLs versus H929; */# p<0.05, **/## p<0.005, ***/### p<0.0005, ns-non-significant; individual p-values provided in the source data file). **(d).** Expression level (mean ± SEM) of ATF6α target genes (*Bip*, *HERPUD1*, *GRP94* and *PDIA4*) in sh-*PLUM* knock down and *PLUM* overexpressed MMCLs (KMS11 and LP1) compared to sh-scramble and vector control respectively (N=3, two-sided unpaired student’s t test; p-values provided in the figure). **(e).** Level of UPR master regulators (p-IRE1α, IRE1α, p-eIF2α, eIF2α and ATF6 α) in NC-ASO, sASO-g4, sASO-g11 and sASO-g12 treated KMS11 cells. (N=3 biological replicates). **(f).** Expression level of UPR master regulators (IRE1α, eIF2α and ATF6α) in sh-IRE1α, sh-eIF2α and sh-AFT6α KD *PLUM* OE KMS11 cells. **(g).** Proliferative rate (mean ± SEM) of sh-eIF2α and sh-AFT6α KD in *PLUM* OE background KMS11 cells. (N=3; two-way ANOVA; VC versus *PLUM* OE: 0.0005, *PLUM*-OE versus sh1-eIF2α: 0.016, *PLUM*-OE versus sh2-eIF2α: 0.0014, *PLUM*-OE versus sh1-ATF6α: 0.0003, *PLUM*-OE versus sh2-ATF6α: 0.001). **(h).** Drug sensitivity IC_50_ curve (mean ± SEM) for sh-eIF2α KD and sh-ATF6α KD in *PLUM* OE background KMS11 cells compared to vector control and scramble RNA in response to BTZ treatment for 24h. (N=2 biological replicates)***.* (i).** Expression level of IRE1α in BTZ-R 8226 and Len-R KMS11 cell lines post shRNA mediated knockdown of IRE1α. (N=2 biological replicates).


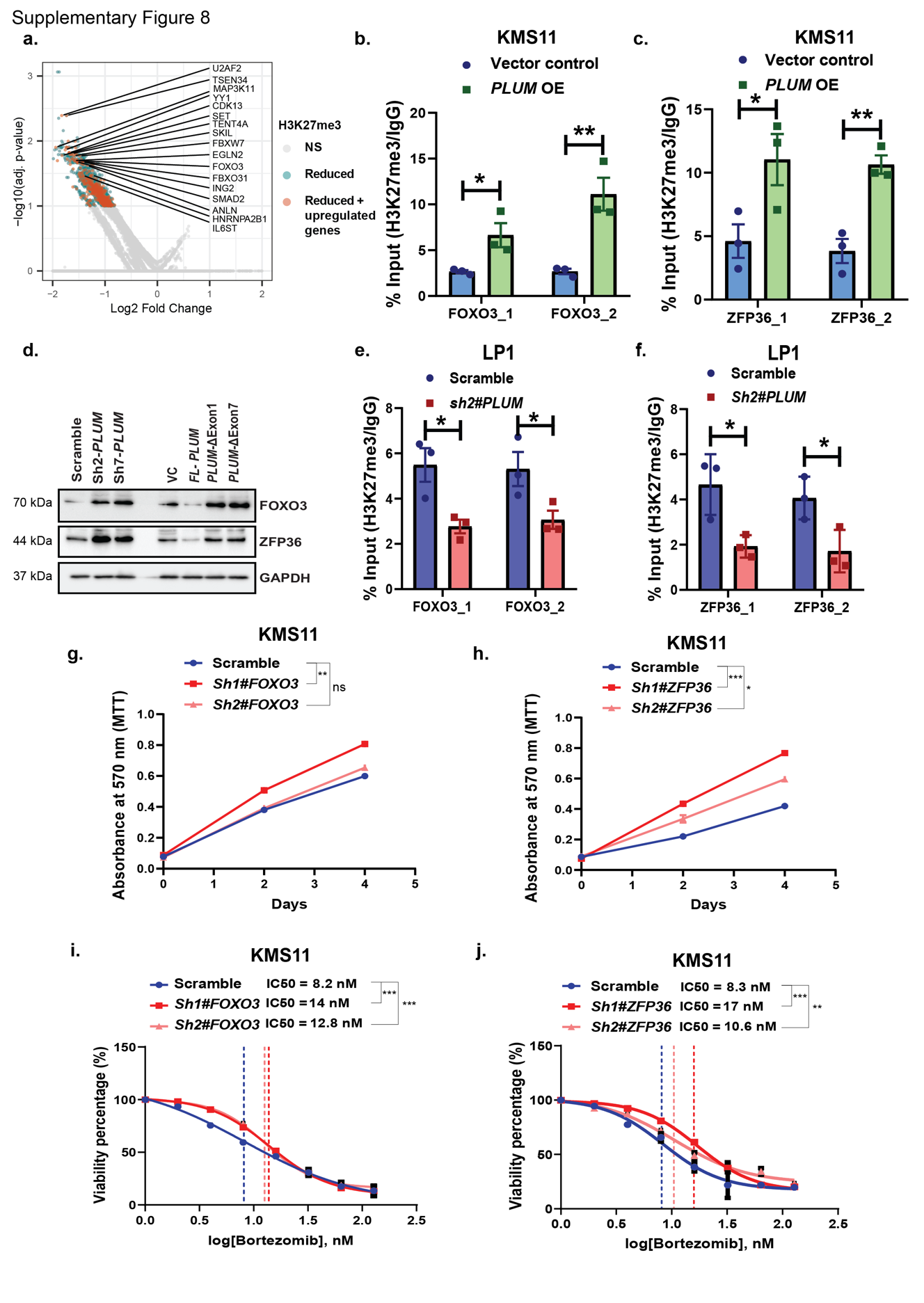
**Supplementary Figure 8: *PLUM*-EZH2 interaction regulates H3K27 methylation marks globally.** (**a)** Scatter plot highlighting significant loss of H3K27me3 at transcription start sites and associated genes upregulated upon *PLUM* KD in KMS11 cells (adj. p-value <= 0.05; NS: non-significant). Genes relevant to enriched biological processes presented are indicated. (**b,c).** ChIP qPCR validation for *FOXO3* and *ZFP36* TSS in VC and FL-*PLUM* overexpressed KMS11 cells (N=3, mean ± SEM is plotted, two-sided unpaired student’s t test; p-values – *FOXO3_1*: 0.04, *FOXO3_2*: 0.009, *ZFP36_1*: 0.05, *ZFP30_2*: 0.004). (**d).** Level of FOXO3 and ZFP36 proteins in *PLUM* KD cells compared to sh-scramble and *PLUM*-FL, *PLUM*-ΔExon1, *PLUM*-ΔExon7 overexpressed cells compared to VC (N=3 biological replicates). (**e,f).** ChIP qPCR validation for *FOXO3* and *ZFP36* TSS in sh-scramble and sh2-*PLUM* knock-down LP1 cells (N=3, mean ± SEM is plotted, two-sided unpaired student’s t test; p-values - *FOXO3_1*: 0.027, *FOXO3_2*: 0.05, *ZFP36_1*: 0.036*, ZFP30_2*: 0.019). (**g).** Proliferative rate (mean ± SEM) of sh-scramble and sh-*FOXO3* knock-down KMS11 cells at time point of 0, 2 and 4 days (N=3, two-way ANOVA done; p-values – scramble versus sh1-*FOXO3*: 0.002, scramble versus sh2-*FOXO3*: non-significant. (**h).** Proliferative rate (mean ± SEM) of sh-scramble and sh-*ZFP36* transduced KMS11 cells at time point of 0, 2 and 4 days (N=3, two-way ANOVA done; p-values – scramble versus sh1-*ZFP36*: 0.0001, scramble versus sh2-*ZFP36*: 0.009. (**i).** Drug sensitivity IC_50_ survival curve (mean ± SEM) of sh-scramble and sh-*FOXO3* KD KMS11 cells post 24 h of treatment with BTZ (N=3, two-way ANOVA; p-values – scramble versus sh1-*FOXO3*: 0.0001, scramble versus sh2-*FOXO3*: 0.0001). (**j).** Drug sensitivity IC_50_ survival curve (mean ± SEM) of sh-scramble and sh-*ZFP36* KD KMS11 cells post 24 h of treatment with BTZ (N=3, two-way ANOVA; p-values – scramble versus sh1-*ZFP36*: 0.0001, scramble versus sh2-*ZFP36*: 0.0037).


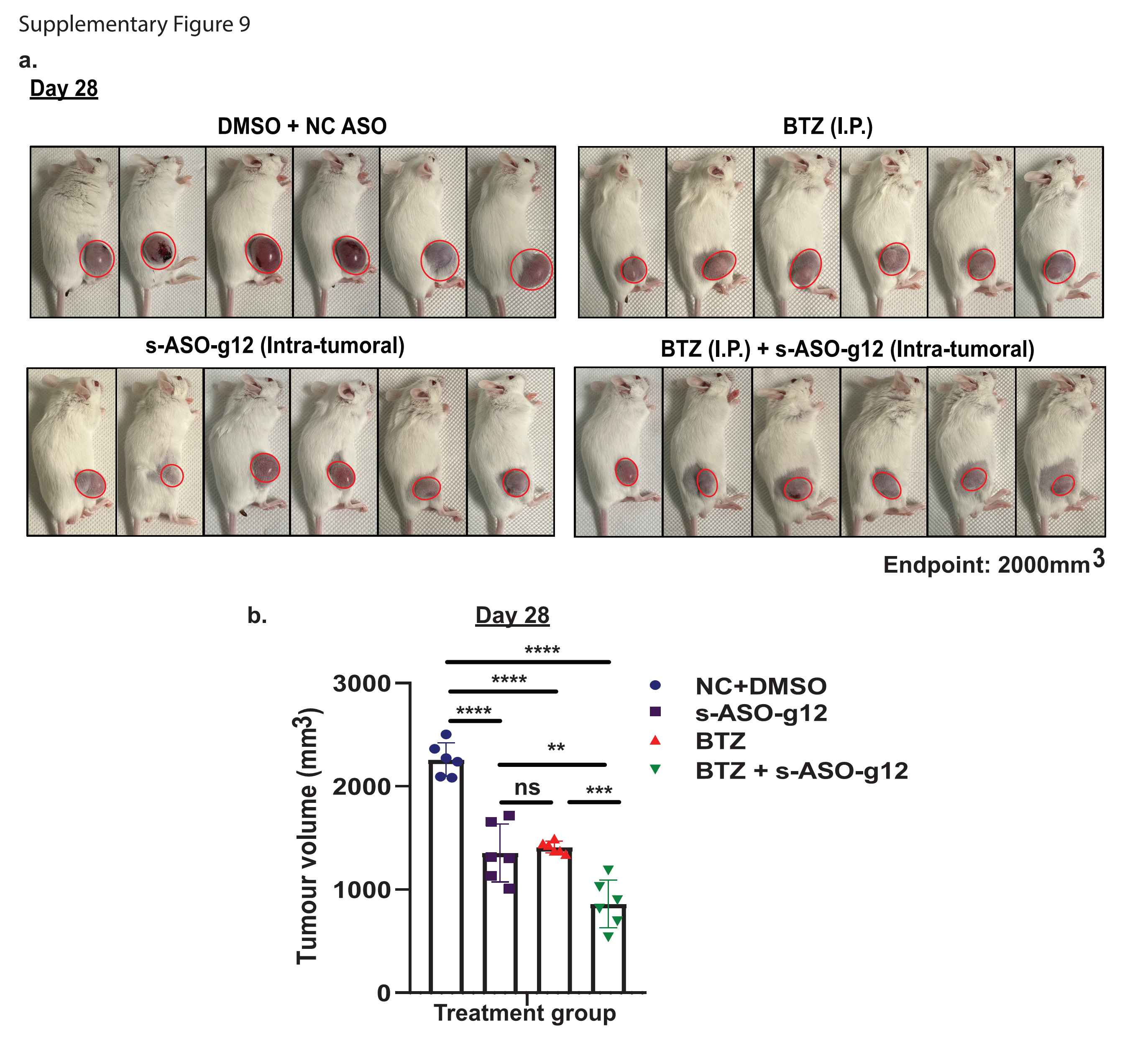
**Supplementary Figure 9: Steric ASO targeted disruption of *PLUM*-EZH2 interaction abrogates the tumour growth and BTZ resistance of MM.** (**a).** Picture showing the *PLUM* overexpressed KMS11 tumor xenografts under different treatment groups (*PLUM* OE + DMSO + NC-ASO, n=6; *PLUM* OE + s-ASO-g12, n=6; *PLUM* OE + BTZ, n=6; *PLUM* OE + s-ASO-g12 + BTZ, n=6) at Day 28. (**b).** Average tumor volume for *PLUM* overexpressed KMS11 xenografts under different treatment groups (*PLUM* OE + DMSO + NC-ASO, n=6; *PLUM* OE + s-ASO-g12, n=6; *PLUM* OE + BTZ, n=6; *PLUM* OE + s-ASO-g12 + BTZ, n=6) at Day 28. (N=6, mean ± SEM is plotted, two-sided unpaired student’s t test; p-values - NC+DMSO versus s-ASO-g12: 0.0001, NC+DMSO versus BTZ: 0.0001, NC+DMSO versus BTZ+s-ASO-g12: 0.0001, s-ASO-g12 versus BTZ: non-significant, s-ASO-g12 versus BTZ+s-ASO-g12: 0.0076, BTZ versus BTZ+s-ASO-g12: 0.0002).

**Supplementary Table 1: List of shRNA sequences used in this study.**

| **Sl No** | **Gene** | **Primer** | **Sequence (5’-3’)** |
| --- | --- | --- | --- |
| 1 | *PLUM* | shRNA2 | GTGCTGGAAGACGGGTATTTA |
|  |  | shRNA7 | AGAAGTATCAGCATCTATTTC |
| 2 | *EZH2* | shRNA1 | CCAACACAAGTCATCCCATTA |
|  |  | shRNA2 | CGGAAATCTTAAACCAAGAAT |
|  |  | shRNA3 | TGTGCCATTGCTAGGTTAATT |
| 3 | *EED* | shRNA1 | GACACTCTGGTGGCAATATTT |
|  |  | shRNA2 | CCTATAACAATGCAGTGTATA |
|  |  | shRNA3 | CCAGTGAATCTAATGTGACTA |
| 4 | *SUZ12* | shRNA1 | GGATGTAAGTTGTCCAATA |
|  |  | shRNA2 | GCTGACAATCAAATGAATCAT |
|  |  | shRNA3 | CGGAATCTCATAGCACCAATA |
| 5 | *IRE1α* | shRNA1 | GCGTAAATTCAGGACCTATAA |
|  |  | shRNA2 | TCAACGCTGGATGGAAGTTTG |
| 6 | *eIF2α* | shRNA1 | GCACCTTCATTTGTTAGATTA |
|  |  | shRNA2 | GCTACTGCTGTGTTGGTAATA |
| 7 | *ATF6α* | shRNA1 | ACAGAGTCTCTCAGGTTAAAT |
|  |  | shRNA2 | GCTTGTCAGTCTCGCAAGA |
| 8 | *FOXO3* | shRNA1 | CCAGAGCCGTCCGCGATCCTG |
|  |  | shRNA2 | GCAAGCACAGAGTTGGATGAA |
|  |  | shRNA3 | AGCACGGTGTTCGACCTTCAT |
| 9 | *ZFP36* | shRNA1 | AACAGAGATGCGATTGAAGA |
|  |  | shRNA2 | GATCCGACCCTGATGAATATG |
|  |  | shRNA3 | GACGGAACTCTGTCACAAGTT |
| 10 | *Scramble* | shRNA | CCTAAGGTTAAGTCGCCCTCG |

**Supplementary Table 2: List of the qPCR primers used in this study.**

| **Sl No** | **Gene** | **Primer sequence (5’-3’)** |
| --- | --- | --- |
| 1 | *PLUM* | F: CCGTGTTGACCAGACTGATT |
|  |  | R: CTCACACCTATAATCTCAGCACTT |
| 2 | *EDEM* | F: CAAGTGTGGGTACGCCACG |
|  |  | R: AAAGAAGCTCTCCATCCGGTC |
| 3 | *GRP94* | F: GAAACGGATGCCTGGTGG |
|  |  | R: GCCCCTTCTTCCTGGGTC |
| 4 | *Bip* | F: TGTTCAACCAATTATCAGCAAACTC |
|  |  | R: TTCTGCTGTATCCTCTTCACCAGT |
| 5 | *CHOP* | F: AGAACCAGGAAACGGAAACAGA |
|  |  | R: TCTCCTTCATGCGCTGCTTT |
| 6 | *ATF4* | F: GTTCTCCAGCGACAAGGCTA |
|  |  | R: ATCCTGCTTGCTGTTGTTGG |
| 7 | *HERPUD1* | F: CCAATGTCTCAGGGACTTGCTTC |
|  |  | R: CGATTAGAACCAGCAGGCTCCT |
| 8 | *PDIA4* | F: AAGCGTTCTCCTCCAATT |
|  |  | R: GGACTGCTCGATCATGTAA |
| 9 | *GAPDH* | F: GCATCCTGGGCTACACTGA |
|  |  | R: CCACCACCCTGTTGCTGTA |

**Supplementary Table 3: List ChIP qPCR primers used in this study.**

| **Sl No** | **Gene** | **Primer sequence (5’-3’)** |
| --- | --- | --- |
| 1 | *FOXO3* | F1: CAGTAAGCAGTCAGAGCCCC |
|  |  | R1: CTGTGTCCTCGCTTCCTTGT |
|  |  | F2: GCACTCTAATGACAAAAGAT |
|  |  | R2: TGGAAGGCTAGATCACCCAG |
| 2 | *ZFP36* | F1: CCCGTGCTTGCAGTTTCCTA |
|  |  | R1: GACTCAGGCATGCAGGTACC |
|  |  | F2: ACTTCAGCGCTCCCACTCTC |
|  |  | R2: GAGTTTGCGGCGCTAGAGAG |


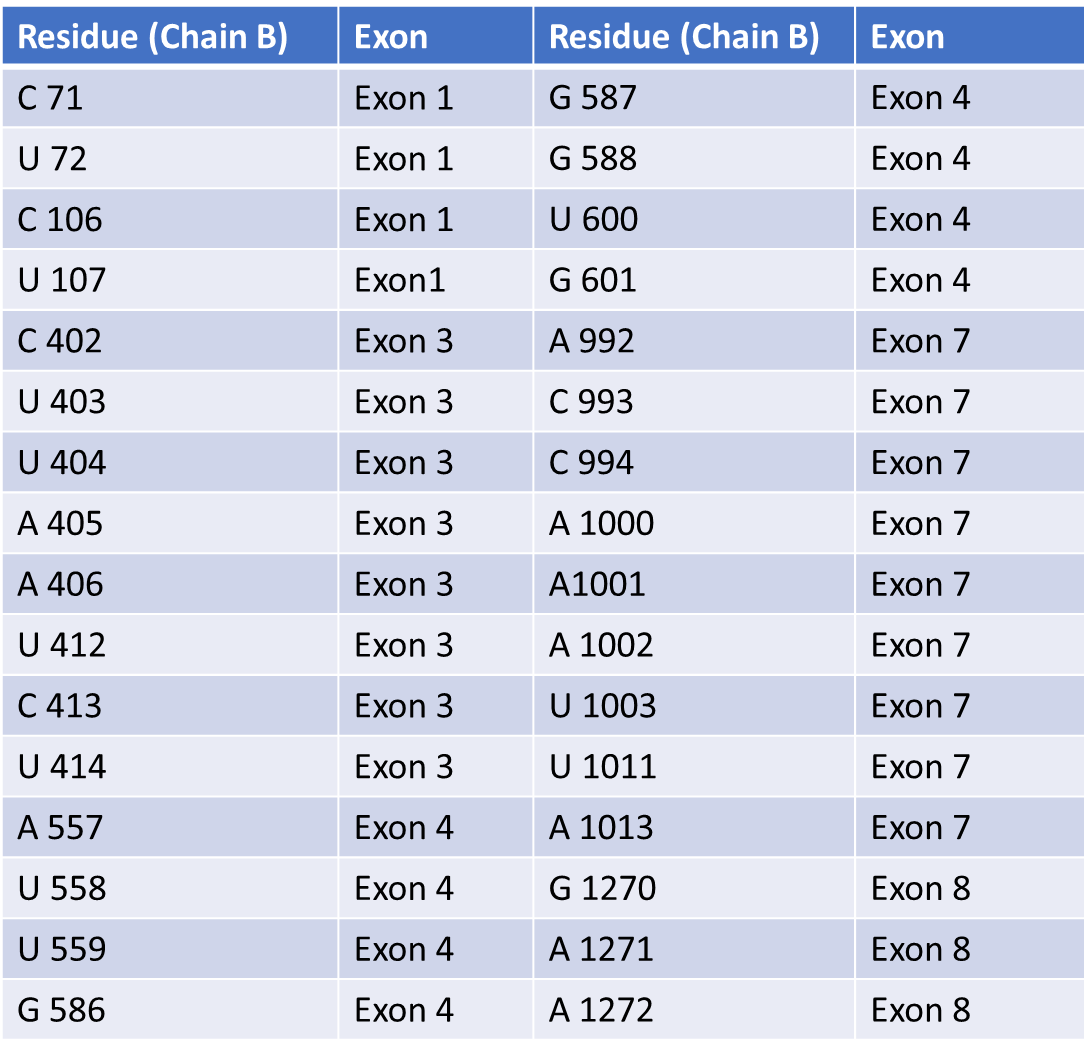
**Supplementary Table 4:** Table showing the residues on chain B (*PLUM*) and its corresponding exon number that are found to interact with EZH2 in the EZH2-*PLUM* complex generated by Alphafold3.

**Supplementary Table 5: List showing the interacting atoms from the RNA (*PLUM*) with 491 to 497 regions on EZH2. The data showed interaction to be with 994, 1000, 1001, 1002, 1003, 1010, 1011 atoms on *PLUM* which are part of its exon 7.**

LYS   491 A CA   | U    1003 B OP1  | 4.84 |    7   1
LYS   491 A CA   | U    1003 B O5'  | 5.37 |    7   2
LYS   491 A C    | U    1003 B O5'  | 5.6  |    6   2
LYS   491 A CB   | U    1003 B C6   | 6.21 |    4   5
LYS   491 A CD   | U    1003 B C5   | 5.78 |    4   5
LYS   491 A CD   | U    1003 B C6   | 5.54 |    4   5
LYS   491 A CE   | U    1003 B OP1  | 5.69 |    7   1
LYS   491 A CE   | U    1003 B O5'  | 5.69 |    7   2
LYS   491 A CE   | U    1003 B O4'  | 5.87 |    7   2
LYS   491 A NZ   | U    1003 B C6   | 6.04 |    3   5
LYS   492 A N    | A    1002 B O3'  | 5.43 |    3   2
LYS   492 A N    | U    1003 B OP1  | 5.03 |    3   1
LYS   492 A N    | U    1003 B O5'  | 5.51 |    3   2
LYS   492 A C    | U    1003 B O5'  | 5.57 |    6   2
LYS   492 A O    | A    1002 B O2'  | 3.77 |    2   1
LYS   492 A CB   | A    1002 B C4'  | 6.18 |    4   8
LYS   493 A N    | U    1003 B O5'  | 5.86 |    3   2
LYS   493 A N    | U    1003 B C5'  | 5.83 |    3   8
LYS   493 A CA   | A    1002 B O2'  | 5.44 |    7   1
LYS   493 A CA   | U    1003 B C5'  | 5.65 |    7   8
LYS   493 A CG   | U    1003 B C5'  | 5.22 |    4   8
LYS   493 A CG   | U    1003 B C5   | 4.06 |    4   5
LYS   493 A CG   | U    1003 B C6   | 4.42 |    4   5
LYS   493 A CD   | U    1003 B C5   | 3.58 |    4   5
LYS   493 A CE   | U    1003 B O4   | 3.5  |    7   2
LYS   493 A CE   | U    1003 B C5   | 2.45 |    7   5
LYS   493 A CE   | U    1003 B C6   | 3.35 |    7   5
LYS   493 A NZ   | U    1003 B C4   | 3.16 |    3   5
LYS   493 A NZ   | U    1003 B O4   | 2.83 |    3   2
LYS   493 A NZ   | U    1003 B C5   | 2.9  |    3   5
ARG   494 A N    | A    1002 B O2'  | 5.16 |    3   1
ARG   494 A CD   | A    1002 B O2'  | 5.26 |    7   1
ARG   494 A CD   | A    1002 B C1'  | 6.05 |    7   6
ARG   494 A NE   | A    1002 B O2'  | 5.68 |    3   1
ARG   494 A NE   | A    1002 B C1'  | 6.13 |    3   6
ARG   494 A CZ   | A    1001 B N3   | 5.52 |    6   3
ARG   494 A CZ   | A    1002 B C1'  | 6.25 |    6   6
ARG   494 A NH1  | A    1000 B C2   | 6.07 |    3   5
ARG   494 A NH1  | A    1001 B C2   | 4.8  |    3   5
LYS   495 A CE   | U    1003 B C4   | 5.76 |    7   5
LYS   495 A CE   | U    1003 B O4   | 5.23 |    7   2
LYS   495 A CE   | U    1003 B C5   | 5.58 |    7   5
LYS   495 A NZ   | U    1003 B O4   | 5.06 |    3   2
ARG   497 A CZ   | U    1010 B O2   | 6.06 |    6   2
ARG   497 A NH1  | U    1011 B O2   | 5.31 |    3   2
ARG   497 A NH2  | C     994 B OP1  | 5.6  |    3   1
ARG   497 A NH2  | U    1011 B C1'  | 5.77 |    3   6
ARG   497 A NH2  | U    1011 B O2   | 4.18 |    3   2

994, 1000, 1001, 1002, 1003, 1010, 1011 (all of these are residues on exon7) which have shown interaction with 491 to 497 regions on EZH2.


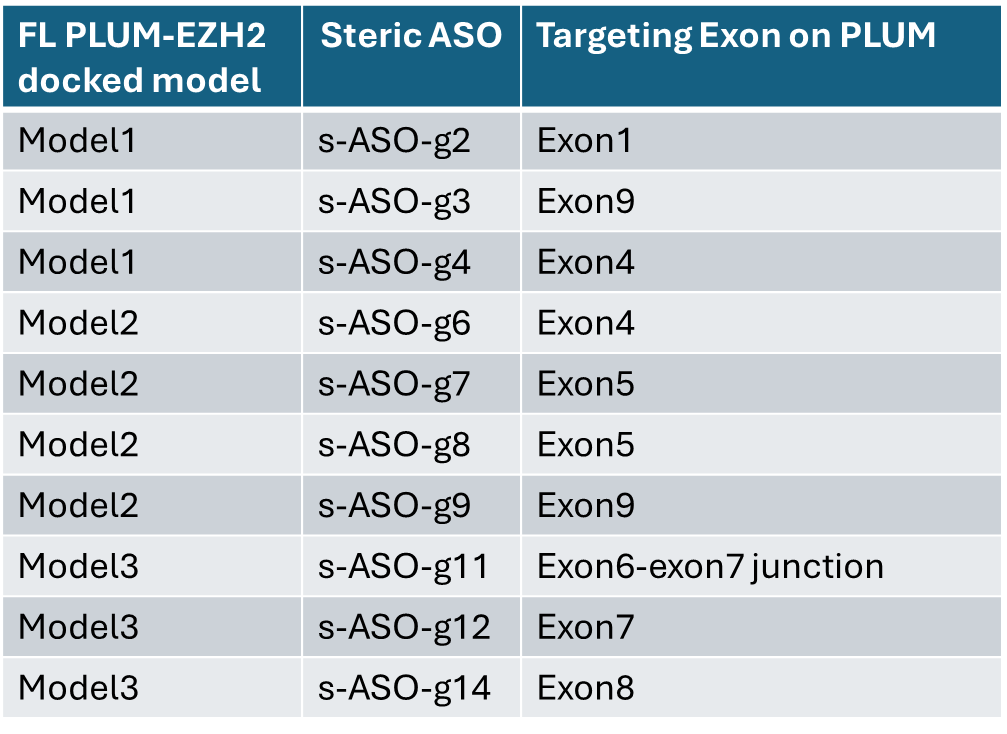
**Supplementary Table 6:** Table showing the designed steric ASOs and their respective targeting exons on *PLUM* based on predicted interacting regions between FL *PLUM*-EZH2 docked models (Model1, Model2 and Model3).

**Supplementary Data 1: Analysed mass-spectrometry data.**

Table showing the LFQ intensity values for each differentially bound protein from two analysis (FL-*PLUM* versus ΔExon1 and FL-*PLUM* versus ΔExon7) supporting our result in Figures 3d and 3e. The table is provided as a separate excel sheet (Supplementary Data 1).

**Statistics:** The MS data has been shortened to the most important MS variables, LFQ intensity values in log2 scale, as well as the log2 fold changes and p-values that were used to create the volcano plot (Figure 3d, 3e). Every single data point on the volcano plot is represented in this list and it may serve as a minimal “Supplementary Table 7”. Differential MS analysis was done with fold change >2 and a p-value < 0.01 (N=4, fold change in LFQ intensity (log2 transformed) on the x-axis and the negative logarithm of the p-value (often base 10) on the y-axis is plotted and statistical significance was determined by unpaired t-test).
