# Supplementary material for: Multiple myeloma associated long non-coding RNA PLUM confers chemoresistance by enhancing PRC2 mediated UPR pathway activation
Source: Nat Commun. 2025 Sep 1;16:8155. doi: 10.1038/s41467-025-63256-x (PMC12402260; doi:10.1038/s41467-025-63256-x)
Supplement: Supplementary file 2 — Description of Additional Supplementary Information [file 41467_2025_63256_MOESM2_ESM.pdf]

Description of Additional Supplementary Files

File Name: Supplementary Data 1
Description: Excel sheet containing raw data for RNA-Protein pull down followed by mass spectrometry. Contains analyzed data for FL PLUM versus PLUM exon1 deletion pull down and FL PLUM versus PLUM exon7 deletion pull down.

File Name: Supplementary Data 2
Description: Excel sheets containing EZH2-PLUM docking interface files.
