# Supplementary material for: Multiple myeloma associated long non-coding RNA PLUM confers chemoresistance by enhancing PRC2 mediated UPR pathway activation
Source: Nat Commun. 2025 Sep 1;16:8155. doi: 10.1038/s41467-025-63256-x (PMC12402260; doi:10.1038/s41467-025-63256-x)
Supplement: Supplementary file 5 — Reporting Summary [file 41467_2025_63256_MOESM5_ESM.pdf]

nature portfolio

Corresponding author(s): Yinghui Li

Last updated by author(s): Jul 10, 2025

Reporting Summary

Nature Portfolio wishes to improve the reproducibility of the work that we publish. This form provides structure for consistency and transparency in reporting. For further information on Nature Portfolio policies, see our Editorial Policies and the Editorial Policy Checklist.

Statistics

For all statistical analyses, confirm that the following items are present in the figure legend, table legend, main text, or Methods section.

n/a	Confirmed	
☐	☒	The exact sample size (*n*) for each experimental group/condition, given as a discrete number and unit of measurement
☐	☒	A statement on whether measurements were taken from distinct samples or whether the same sample was measured repeatedly
☐	☒	The statistical test(s) used AND whether they are one- or two-sided<br>*Only common tests should be described solely by name; describe more complex techniques in the Methods section.*
☐	☒	A description of all covariates tested
☐	☒	A description of any assumptions or corrections, such as tests of normality and adjustment for multiple comparisons
☐	☒	A full description of the statistical parameters including central tendency (e.g. means) or other basic estimates (e.g. regression coefficient) AND variation (e.g. standard deviation) or associated estimates of uncertainty (e.g. confidence intervals)
☐	☒	For null hypothesis testing, the test statistic (e.g. *F*, *t*, *r*) with confidence intervals, effect sizes, degrees of freedom and *P* value noted<br>*Give P values as exact values whenever suitable.*
☒	☐	For Bayesian analysis, information on the choice of priors and Markov chain Monte Carlo settings
☒	☐	For hierarchical and complex designs, identification of the appropriate level for tests and full reporting of outcomes
☒	☐	Estimates of effect sizes (e.g. Cohen's *d*, Pearson's *r*), indicating how they were calculated

Our web collection on statistics for biologists contains articles on many of the points above.

Software and code

Policy information about availability of computer code

Data collection	qPCR and ChIP qPCR: CFX Maestro Software<br>Western blots/gel analysis: ImageJ<br>Bioanalyser: Agilent 2100 Bioanalyser software<br>FACS (Apoptosis): BD FACSDiva™/Flowjo Software<br>Colorimetric analysis: Tecan icontrol<br>Mass spectrometry: EASY-nLC 1200 System (Thermo)
Data analysis	#mass spectrometry<br>MaxQuant version 1.5.2.8<br>#RNA structure prediction and Docking<br>3dRNA v2.0: http://biophy.hust.edu.cn/new/3dRNA<br>RNAComposer: https://rnacomposer.cs.put.poznan.pl/about<br>rsRNASP: https://github.com/Tan-group/rsRNASP<br>cgRNASP: https://github.com/Tan-group/cgRNASP<br>I-TASSER Suite 5.2: https://zhanggroup.org/I-TASSER/<br>HDOCK: http://hdock.phys.hust.edu.cn/<br><br>#bash

bedtools 2.30.0
bowtie2 2.3.4.3
macs2 2.1.2
#R
biomaRt 2.50.0
ChIPseqSpikeInFree_1.2.4
circlize 0.4.15
ClusterProfiler 4.2.0
ComplexHeatmap 2.10.0
DESeq2 1.34.0
DOSE 3.20.0
edgeR 3.36.0
eulerr 6.1.1
factoextra 1.0.7
ggplot2 3.3.5
GO.db 3.14.0

For manuscripts utilizing custom algorithms or software that are central to the research but not yet described in published literature, software must be made available to editors and reviewers. We strongly encourage code deposition in a community repository (e.g. GitHub). See the Nature Portfolio guidelines for submitting code & software for further information.

Data

Policy information about availability of data

All manuscripts must include a data availability statement. This statement should provide the following information, where applicable:

- Accession codes, unique identifiers, or web links for publicly available datasets
- A description of any restrictions on data availability
- For clinical datasets or third party data, please ensure that the statement adheres to our policy

The mass spectrometry data have been deposited to the ProteomeXchange Consortium via the PRIDE partner repository with the dataset identifier PXD054586. The analysed mass spectrometry data is available in supplementary file 3. Sequencing data are available at GEO under accession numbers GSE230526 (https://www.ncbi.nlm.nih.gov/geo/query/acc.cgi?acc=GSE230526) and GSE274152 (https://www.ncbi.nlm.nih.gov/geo/query/acc.cgi?acc=GSE274152). The p52 knockdown RNA-seq data used in this study are available in the GEO database under accession code GSE230293 (https://www.ncbi.nlm.nih.gov/geo/query/acc.cgi?acc=GSE230293).
All MMRF CoMMpass data can be accessed at https://research.themmrf.org/
CCLE dataset can be accessed at https://sites.broadinstitute.org/ccle/datasets

Research involving human participants, their data, or biological material

Policy information about studies with human participants or human data. See also policy information about sex, gender (identity/presentation), and sexual orientation and race, ethnicity and racism.

Reporting on sex and gender	*Use the terms sex (biological attribute) and gender (shaped by social and cultural circumstances) carefully in order to avoid confusing both terms. Indicate if findings apply to only one sex or gender; describe whether sex and gender were considered in study design; whether sex and/or gender was determined based on self-reporting or assigned and methods used. Provide in the source data disaggregated sex and gender data, where this information has been collected, and if consent has been obtained for sharing of individual-level data; provide overall numbers in this Reporting Summary. Please state if this information has not been collected. Report sex- and gender-based analyses where performed, justify reasons for lack of sex- and gender-based analysis.*
Reporting on race, ethnicity, or other socially relevant groupings	*Please specify the socially constructed or socially relevant categorization variable(s) used in your manuscript and explain why they were used. Please note that such variables should not be used as proxies for other socially constructed/relevant variables (for example, race or ethnicity should not be used as a proxy for socioeconomic status). Provide clear definitions of the relevant terms used, how they were provided (by the participants/respondents, the researchers, or third parties), and the method(s) used to classify people into the different categories (e.g. self-report, census or administrative data, social media data, etc.) Please provide details about how you controlled for confounding variables in your analyses.*
Population characteristics	*Describe the covariate-relevant population characteristics of the human research participants (e.g. age, genotypic information, past and current diagnosis and treatment categories). If you filled out the behavioural & social sciences study design questions and have nothing to add here, write "See above."*
Recruitment	*Describe how participants were recruited. Outline any potential self-selection bias or other biases that may be present and how these are likely to impact results.*
Ethics oversight	*Identify the organization(s) that approved the study protocol.*

Note that full information on the approval of the study protocol must also be provided in the manuscript.

Field-specific reporting

Please select the one below that is the best fit for your research. If you are not sure, read the appropriate sections before making your selection.

☒ Life sciences ☐ Behavioural & social sciences ☐ Ecological, evolutionary & environmental sciences

For a reference copy of the document with all sections, see nature.com/documents/nr-reporting-summary-flat.pdf

Life sciences study design

All studies must disclose on these points even when the disclosure is negative.

Sample size

No sample size calculation was performed. Sample sizes followed prevalent guidelines for balancing statistical power/cost and expert advice.

Data exclusions

No data were deliberately excluded.

Replication

Endogenous HA-EZH2 ChIP seq:
Reproducible EZH2 binding were consistently identified through independent ChIP seq experiments (n≥2)

Scramble and sh-PLUM (KMS11) H3K27me3 ChIP seq:
Reproducible KMS11 H3K27 tri-methylation changes were consistently measured by H3K27me3 ChIP seq across three biological replicates per condition. Efficiency of PLUM KD was assessed prior to sequencing by qPCR.

BTZ resistant versus BTZ sensitive (R8226) H3K27me3 ChIP qPCR:
Reproducible R8226-BTZ resistant and R8226-BTZ sensitive H3K27 tri-methylation changes were consistently measured by H3K27me3 ChIP-qPCR for target genes (ZFP36 and FOXO3) across three biological replicates per condition.

Len resistant versus Len sensitive (KMS11) H3K27me3 ChIP qPCR:
Reproducible KMS11-Len resistant and KMS11-Len sensitive H3K27 tri-methylation changes were consistently measured by H3K27me3 ChIP-qPCR for target genes (ZFP36 and FOXO3) across three biological replicates per condition.

BTZ resistant-R8226 (+/- s-ASO-g12 treated) H3K27me3 ChIP qPCR:
Reproducible BTZ resistant (+/- s-ASO-g12 treated) R8226 cell line H3K27 tri-methylation changes were consistently measured by H3K27me3 ChIP-qPCR for target genes (ZFP36 and FOXO3) across three biological replicates per condition.

Len resistant-KMS11 (+/- s-ASO-g12 treated) H3K27me3 ChIP qPCR:
Reproducible Len resistant (+/- s-ASO-g12 treated) KMS11 cell line H3K27 tri-methylation changes were consistently measured by H3K27me3 ChIP-qPCR for target genes (ZFP36 and FOXO3) across three biological replicates per condition.

RNA-protein pull down + mass spectrometry (Full length PLUM, Δ Exon1 PLUM and Δ Exon7 PLUM with nuclear lysate of KMS11):
The differentially bound RNA binding proteins (RBPs) to each of the in-vitro transcribed transcripts were consistently identified by mass spectrometry analysis across 4 biological replicates per condition.

Mouse experiments :
BTZ drug response study - Reproducible in-vivo phenotypes were consistently measured across treatment groups consisting of 5 animals.
ASO and BTZ conbination drug response study - Reproducible in-vivo phenotypes were consistently measured across treatment groups consisting of 6 animals.

Western blots: Blots shown are a representation of ≥ 2 replicates.
RIP-qPCR: It was done with ≥ 2 biological replicates.
RNA-FISH and RNA-FISH/IF: Images were captured with ≥ 4 frames per condition.
Functional assays: Apoptosis assay staining was done with ≥ 2 biological replicates per condition. Proliferation assay (MTT assay) was done with 3 biological replicates per condition. Drug sensitivity curve assay was done with 3 biological replicates per condition.

Randomization

Randomization was only relevant to in-vivo experiments where mice were randomly allocated into control and treatment groups irrespective of weight, size and gender. The in-vitro experiments are exploratory in nature where experiments were designed with fixed known conditions to observe reproducibility.

Blinding

Blinding was not necessary throughout the study due to the objective nature of the measurements taken.

Behavioural & social sciences study design

All studies must disclose on these points even when the disclosure is negative.

Study description

Briefly describe the study type including whether data are quantitative, qualitative, or mixed-methods (e.g. qualitative cross-sectional, quantitative experimental, mixed-methods case study).

Research sample	*State the research sample (e.g. Harvard university undergraduates, villagers in rural India) and provide relevant demographic information (e.g. age, sex) and indicate whether the sample is representative. Provide a rationale for the study sample chosen. For studies involving existing datasets, please describe the dataset and source.*
Sampling strategy	*Describe the sampling procedure (e.g. random, snowball, stratified, convenience). Describe the statistical methods that were used to predetermine sample size OR if no sample-size calculation was performed, describe how sample sizes were chosen and provide a rationale for why these sample sizes are sufficient. For qualitative data, please indicate whether data saturation was considered, and what criteria were used to decide that no further sampling was needed.*
Data collection	*Provide details about the data collection procedure, including the instruments or devices used to record the data (e.g. pen and paper, computer, eye tracker, video or audio equipment) whether anyone was present besides the participant(s) and the researcher, and whether the researcher was blind to experimental condition and/or the study hypothesis during data collection.*
Timing	*Indicate the start and stop dates of data collection. If there is a gap between collection periods, state the dates for each sample cohort.*
Data exclusions	*If no data were excluded from the analyses, state so OR if data were excluded, provide the exact number of exclusions and the rationale behind them, indicating whether exclusion criteria were pre-established.*
Non-participation	*State how many participants dropped out/declined participation and the reason(s) given OR provide response rate OR state that no participants dropped out/declined participation.*
Randomization	*If participants were not allocated into experimental groups, state so OR describe how participants were allocated to groups, and if allocation was not random, describe how covariates were controlled.*

Ecological, evolutionary & environmental sciences study design

All studies must disclose on these points even when the disclosure is negative.

Study description	*Briefly describe the study. For quantitative data include treatment factors and interactions, design structure (e.g. factorial, nested, hierarchical), nature and number of experimental units and replicates.*
Research sample	*Describe the research sample (e.g. a group of tagged Passer domesticus, all Stenocereus thurberi within Organ Pipe Cactus National Monument), and provide a rationale for the sample choice. When relevant, describe the organism taxa, source, sex, age range and any manipulations. State what population the sample is meant to represent when applicable. For studies involving existing datasets, describe the data and its source.*
Sampling strategy	*Note the sampling procedure. Describe the statistical methods that were used to predetermine sample size OR if no sample-size calculation was performed, describe how sample sizes were chosen and provide a rationale for why these sample sizes are sufficient.*
Data collection	*Describe the data collection procedure, including who recorded the data and how.*
Timing and spatial scale	*Indicate the start and stop dates of data collection, noting the frequency and periodicity of sampling and providing a rationale for these choices. If there is a gap between collection periods, state the dates for each sample cohort. Specify the spatial scale from which the data are taken*
Data exclusions	*If no data were excluded from the analyses, state so OR if data were excluded, describe the exclusions and the rationale behind them, indicating whether exclusion criteria were pre-established.*
Reproducibility	*Describe the measures taken to verify the reproducibility of experimental findings. For each experiment, note whether any attempts to repeat the experiment failed OR state that all attempts to repeat the experiment were successful.*
Randomization	*Describe how samples/organisms/participants were allocated into groups. If allocation was not random, describe how covariates were controlled. If this is not relevant to your study, explain why.*
Blinding	*Describe the extent of blinding used during data acquisition and analysis. If blinding was not possible, describe why OR explain why blinding was not relevant to your study.*

Did the study involve field work? ☐ Yes ☒ No

Reporting for specific materials, systems and methods

We require information from authors about some types of materials, experimental systems and methods used in many studies. Here, indicate whether each material, system or method listed is relevant to your study. If you are not sure if a list item applies to your research, read the appropriate section before selecting a response.

Materials & experimental systems

n/a	Involved in the study
	x Antibodies
	x Eukaryotic cell lines
x	Palaeontology and archaeology
	x Animals and other organisms
x	Clinical data
x	Dual use research of concern
x	Plants

Methods

n/a	Involved in the study
	x ChIP-seq
	x Flow cytometry
x	MRI-based neuroimaging

Antibodies

Antibodies used

Antibodies for western blotting: NFKB2 (1:1000; 3017, CST), p-EZH2 (1:1000; PA5114574, Thermo), EZH2 (1:1000; 491043, Thermo), EED (1:1000; PA534430, Thermo), SUZ12 (1:1000; 3737S, CST), p-IRE1α (1:1000; ab243665, Abcam), p21/CDKN1A (1:1000; 2947S, CST), p15/CDKN2B (1:1000; 36303S, CST), p14/CDKN2A (1:500; sc-53639, santacruz), IRE1α (1:500; sc-390960, santacruz), sXBP1 (1:500; sc-8015, santacruz), p-eIF2α (1:1000; 9721, CST), eIF2α (1:500; sc-133132, santacruz), ATF6α (1:500; sc-166659, santacruz), GAPDH (1:10000; sc-32233, santacruz), HA antibody (1:1000; sc-7392, santacruz), pCDK1/2 (1:1000; 4539S, CST), CDK1/2 (1:500; sc-53219, santacruz), H3K27me3 (1:1000; 9733S, CST), H3 (1:1000; sc-517576, santacruz), FKHRL1/FOXO3 (1:500; sc-48348, santacruz), TTP/ZFP36 (1:500; sc-374305, santacruz), anti-rabbit IgG-HRP conjugated secondary antibody (1:10000; sc-2357, santacruz) and anti-mouse IgG-HRP conjugated secondary antibody (1:10000; sc-516102, santacruz).

Antibodies for ChIP: H3K27me3 (9733S; CST), EZH2 (491043; Life technologies), HA Ab (901503; Genomax), Anti-rabbit IgG (7074S; CST).

Validation

NFKB2 (3017; CST): Western blot of extracts from HeLa and COS cells, using NF-kB2 p100/p52 (18D10) Rabbit mAb.
Reactivity: Human, Monkey. Sensitivity: Endogenous. Source/Isotype: Rabbit IgG.
p-EZH2 (PA5114574;Thermo): Western blot of extracts from mouse brain and VERO cells, using pEZH2 (Thr345) rabbit polyclonal antibody. Reactivity: Human, Mouse, non-human primate, Rat. Sensitivity: phosphorylated EZH2 at Thr345 site. Source/Isotype: Rabbit IgG.
EZH2 (491043;Thermo): Western blot of extracts from PC3, MCF7, T98G, DU145, HCT116 and HeLa cells, using EZH2 (49-1043) rabbit polyclonal antibody. Reactivity: Human, Mouse. Sensitivity: N-terminus (aa1-343) of the mouse Ezh2 protein. Source/Isotype: Rabbit IgG.
EED (PA534430;Thermo): Western blot of extracts from A549 cells, rat kidney tissue, mouse lung tissue, mouse spleen tissue, mouse pancreas tissue, human heart tissue, human testis tissue and CG tissue using EED rabbit polyclonal antibody. Reactivity: Human, Mouse, Rat. Sensitivity: Endogenous. Source/Isotype: Rabbit IgG.
SUZ12 (3737S;CST): Western blot of extracts from HeLa, F9, COS and C6 cells using SUZ12 (D39F6) rabbit mAb. Reactivity: Human, Mouse, Rat, Monkey. Sensitivity: Endogenous. Source/Isotype: Rabbit IgG.
p-IRE1α (ab243665;Abcam): Western blot of extracts from HeLa cells using Anti-IRE1 (phospho S724) rabbit mAb. Reactivity: Human. Sensitivity: Phospho IRE1 (S724). Source/Isotype: Rabbit IgG.
p21/CDKN1A (2947S; CST): Western blot of extracts from HeLa, HUVEC, COS, SH-SY5Y and MCF7 cells using p21 Waf1/Cip1 (12D1) Rabbit mAb. Reactivity: Human, Monkey. Sensitivity: Endogenous. Source/Isotype: Rabbit IgG.
p15/CDKN2B (36303S; CST): Western blot of extracts from HaCaT cells using p15 INK4B (E3R6S) Rabbit mAb. Reactivity: Human. Sensitivity: Endogenous. Source/Isotype: Rabbit IgG.
p14/CDKN2A (sc-53639; santacruz): Western blot of extracts from DU145, HeLa and BJAB cells using p14ARF Antibody (DCS-240) mouse mAb. p14 ARF (DCS-240) is a mouse monoclonal antibody raised against the N-terminus of p14 ARF of human origin. Source/Isotype: Mouse IgG.
IRE1α (sc-390960;Santacruz): Western blot of extracts from DU145, Jurkat, HeLa, KNRK and C2C12 cells using IRE1α Antibody (B-12) mouse mAb. It is raised against amino acids 371-560 of IRE1α of human origin. Reactivity: Human, Mouse and Rat. Source/Isotype: Mouse IgG.
sXBP1 (sc-8015; santacruz): Western blot of exogenously expressed fusion protein using XBP1 Antibody (F-4) mouse mAb. It is raised against amino acids 76-263 mapping at the C-terminus of XBP-1 of mouse origin. Reactivity: Human, Mouse and Rat. Source/Isotype: Mouse IgG.
p-eIF2α (9721; CST): Western blot of extracts from PC12 and C2C12 cells using Phospho-eIF2α (Ser51) Rabbit mAb. Reactivity: Human, Mouse, Rat, Monkey, Donkey. Sensitivity: Endogenous. Source/Isotype: Rabbit IgG.
eIF2α (sc-133132;santacruz): Western blot of extracts from HeLa, PC12, A-431, NIH/3T3, KNRK, C6 and Jurkat cells using eIF2α Antibody (D-3) mouse mAb. It is raised against acids 1-315 representing full length eIF2α of human origin. Reactivity: Human, Mouse and Rat. Source/Isotype: Mouse IgG.
ATF6α (sc-166659;santacruz): Western blot of extracts from MIA-PaCa-2, MDA-MB231, C4, MCF7 cells, rat liver tissue and human testis tissue using ATF-6α (F-7) mouse mAb. It is raised against amino acids 31-310 of ATF-6 of human origin. Reactivity: Human, Mouse and Rat. Source/Isotype: Mouse IgG.
GAPDH (sc-32233;santacruz): Western blot of extracts from HepG2, A549, Raji, PC-3, U-251-MG, HeLa, C4, MOLT-4 and Jurkat cells using GAPDH (6C5) mouse mAb. It is raised against GAPDH purified from rabbit muscle. Reactivity: Human, Mouse, Rat, Rabbit and Xenopus origin. Source/Isotype: Mouse IgG.
pCDK1/2 or pCDC2 (4539S;CST): Western blot of extracts from C6 and HeLa cells using Phospho-cdc2 (Tyr15) (10A11) Rabbit mAb. Reactivity: Human, Mouse, Rat, Monkey. Sensitivity: Endogenous. Source/Isotype: Rabbit IgG.
CDK1/2 (sc-53219;santacruz): Western blot of extracts from K562, HeLa, NAMALWA, CCRF-CEM, Jurkat and TK-1 cells using Cdk1/

Cdk2 (AN21.2) mouse mAb. It is raised against human recombinant Cdk2. Reactivity: Human, Mouse, Rat. Source/Isotype: Mouse IgG.
H3K27me3 (9733S; CST): Western blot of extracts from HCT-116, NIH-3T3, C6 and COS cells using Tri-Methyl-Histone H3 (Lys27) (C36B11) Rabbit mAb. Reactivity: Human, Mouse, Rat, Monkey. Sensitivity: Endogenous. Source/Isotype: Rabbit IgG.
H3 (sc-517576; santacruz): It is raised against recombinant Histone H3 protein fragments of human origin. Reactivity: Human, Mouse, Rat. Source/Isotype: Mouse IgG.
FKHRL1/FOXO3 (sc-48348;santacruz): Western blot of extracts from MDA-MB231, A-673, RD, SJRH30, TF-1, NCI-H929, TE671, HeLa, human adrenal gland tissue, A375, F-9 and 293T cells using FKHRL1/FOXO3a (D-12) mouse mAb. It is raised against amino acids 329-472 of FKHRL1 of human origin. Reactivity: Human, Mouse, Rat. Source/Isotype: Mouse IgG.
TTP/ZFP36 (sc-374305;santacruz): Western blot of extracts from K562, A431, Jurkat, RAW309 Cr.1 and 293T cells using TTP (A-8) mouse mAb. It is raised against amino acids 166-285 mapping near the C-terminus of TTP of human origin. Reactivity: Human, Mouse, Rat. Source/Isotype: Mouse IgG.

H3K27me3 (9733S; CST): Tri-Methyl-Histone H3 (Lys27) (C36B11) Rabbit mAb detects endogenous levels of histone H3 only when tri-methylated on Lys27. The antibody does not cross-react with non-methylated, mono-methylated or di-methylated Lys27. In addition, the antibody does not cross-react with mono-methylated, di-methylated or tri-methylated histone H3 at Lys4, Lys9, Lys36 or Histone H4 at Lys20. This antibody has been validated using SimpleChIP® Enzymatic Chromatin IP Kits. Reactivity: Human, Mouse, Rat, Monkey. Host: Rabbit monoclonal antibody IgG.

HA Ab (901503; Genomax): Monoclonal antibody HA.11 was raised against the twelve amino acid peptide CYPYDVPDYASL. The HA.11 antibody recognizes HA epitopes located in the middle of protein sequences as well as at the N- or C-terminus. Host: Mouse monoclonal antibody IgG1, k isotype.

Eukaryotic cell lines

Policy information about cell lines and Sex and Gender in Research

Cell line source(s)	KMS11 (female), XG-7 (female), H929 (female), JJN3 (female), RPMI8226 (male) and U266 (male) were kind gift from Prof. Leif Bergsagel (Mayo Clinic, Scottsdale, AZ, USA). The MM1.S (female) cell line was obtained from ATCC while LP1 (female) and MOLP8 (male) were obtained from the German Collection of Microorganisms and Cell Cultures. RPMI 8226 BTZ resistant, KMS11 lenalidomide resistant and their parent sensitive cells were obtained from the lab of Prof Wee Joo Chng (Cancer Science Institute, NUS, Singapore).
Authentication	Cell lines were authenticated using STR (Applied Biosystems)
Mycoplasma contamination	All cell lines were tested to be mycoplasma-free using Mycoplasma PCR Detection Kit (G238; Abm) prior to experiments.
Commonly misidentified lines (See ICLAC register)	According to the ICLAC, H929 (NCI-H9292) has been reported to be contaminated with K-562 cells. However, authentic stock is known to exist. Our stocks have been recently authenticated by STR analysis (Centre for Translational Research and Diagnostics, National University of Singapore).

Palaeontology and Archaeology

Specimen provenance	*Provide provenance information for specimens and describe permits that were obtained for the work (including the name of the issuing authority, the date of issue, and any identifying information). Permits should encompass collection and, where applicable, export.*
Specimen deposition	*Indicate where the specimens have been deposited to permit free access by other researchers.*
Dating methods	*If new dates are provided, describe how they were obtained (e.g. collection, storage, sample pretreatment and measurement), where they were obtained (i.e. lab name), the calibration program and the protocol for quality assurance OR state that no new dates are provided.*

☐ Tick this box to confirm that the raw and calibrated dates are available in the paper or in Supplementary Information.

Ethics oversight	*Identify the organization(s) that approved or provided guidance on the study protocol, OR state that no ethical approval or guidance was required and explain why not.*

Note that full information on the approval of the study protocol must also be provided in the manuscript.

Animals and other research organisms

Policy information about studies involving animals; ARRIVE guidelines recommended for reporting animal research, and Sex and Gender in Research

Laboratory animals	Xenograft experiment: Balb/c RAG -/- IL2Rγ -/- mice (6-10 weeks old) were used.
Wild animals	No wild animals were used in this study.

Reporting on sex	sex was not considered in this study design and each treatment condition had a mixture of male and female mice.
Field-collected samples	No field collected samples were used in this study.
Ethics oversight	Xenograft experiments: All studies were approved by Institutional Animal Care and Use Committee of Nanyang Technological University, Singapore (NTU-ARF; AUP: A21070).

Note that full information on the approval of the study protocol must also be provided in the manuscript.

Clinical data

Policy information about clinical studies

All manuscripts should comply with the ICMJEguidelines for publication of clinical research and a completedCONSORT checklist must be included with all submissions.

Clinical trial registration	*Provide the trial registration number from ClinicalTrials.gov or an equivalent agency.*
Study protocol	*Note where the full trial protocol can be accessed OR if not available, explain why.*
Data collection	*Describe the settings and locales of data collection, noting the time periods of recruitment and data collection.*
Outcomes	*Describe how you pre-defined primary and secondary outcome measures and how you assessed these measures.*

Dual use research of concern

Policy information about dual use research of concern

Hazards

Could the accidental, deliberate or reckless misuse of agents or technologies generated in the work, or the application of information presented in the manuscript, pose a threat to:

No	Yes	
x		Public health
x		National security
x		Crops and/or livestock
x		Ecosystems
x		Any other significant area

Experiments of concern

Does the work involve any of these experiments of concern:

No	Yes	
x		Demonstrate how to render a vaccine ineffective
x		Confer resistance to therapeutically useful antibiotics or antiviral agents
x		Enhance the virulence of a pathogen or render a nonpathogen virulent
x		Increase transmissibility of a pathogen
x		Alter the host range of a pathogen
x		Enable evasion of diagnostic/detection modalities
x		Enable the weaponization of a biological agent or toxin
x		Any other potentially harmful combination of experiments and agents

Plants

Seed stocks

Report on the source of all seed stocks or other plant material used. If applicable, state the seed stock centre and catalogue number. If plant specimens were collected from the field, describe the collection location, date and sampling procedures.

Novel plant genotypes

Describe the methods by which all novel plant genotypes were produced. This includes those generated by transgenic approaches, gene editing, chemical/radiation-based mutagenesis and hybridization. For transgenic lines, describe the transformation method, the number of independent lines analyzed and the generation upon which experiments were performed. For gene-edited lines, describe the editor used, the endogenous sequence targeted for editing, the targeting guide RNA sequence (if applicable) and how the editor was applied.

Authentication

Describe any authentication procedures for each seed stock used or novel genotype generated. Describe any experiments used to assess the effect of a mutation and, where applicable, how potential secondary effects (e.g. second site T-DNA insertions, mosiacism, off-target gene editing) were examined.

ChIP-seq

Data deposition

☒ Confirm that both raw and final processed data have been deposited in a public database such as GEO.

☒ Confirm that you have deposited or provided access to graph files (e.g. BED files) for the called peaks.

Data access links
May remain private before publication.

GSE274152
https://www.ncbi.nlm.nih.gov/geo/query/acc.cgi?acc=GSE274152

Files in database submission

H3K27me3_KMS11_plumKD_ctrl_rep2.bw
EZH2_KMS11_endogenous_ctrl_rep1.bw
EZH2_KMS11_endogenous_ctrl_rep1.narrowPeak.gz
EZH2_KMS11_endogenous_ctrl_rep1_1.fq.gz
EZH2_KMS11_endogenous_ctrl_rep1_2.fq.gz
EZH2_KMS11_endogenous_ctrl_rep2.bw
EZH2_KMS11_endogenous_ctrl_rep2.narrowPeak.gz
EZH2_KMS11_endogenous_ctrl_rep2_1.fq.gz
EZH2_KMS11_endogenous_ctrl_rep2_2.fq.gz
EZH2_KMS11_endogenous_ctrl_rep3.bw
EZH2_KMS11_endogenous_ctrl_rep3.narrowPeak.gz
EZH2_KMS11_endogenous_ctrl_rep3_1.fq.gz
EZH2_KMS11_endogenous_ctrl_rep3_2.fq.gz
H3K27me3_KMS11_plumKD_ctrl_rep1.bw
H3K27me3_KMS11_plumKD_ctrl_rep1.narrowPeak.gz
H3K27me3_KMS11_plumKD_ctrl_rep1_1.fq.gz
H3K27me3_KMS11_plumKD_ctrl_rep1_2.fq.gz
H3K27me3_KMS11_plumKD_ctrl_rep2.narrowPeak.gz
H3K27me3_KMS11_plumKD_ctrl_rep2_1.fq.gz
H3K27me3_KMS11_plumKD_ctrl_rep2_2.fq.gz
H3K27me3_KMS11_plumKD_ctrl_rep3.bw
H3K27me3_KMS11_plumKD_ctrl_rep3.narrowPeak.gz
H3K27me3_KMS11_plumKD_ctrl_rep3_1.fq.gz
H3K27me3_KMS11_plumKD_ctrl_rep3_2.fq.gz
H3K27me3_KMS11_plumKD_trgt_rep1.bw
H3K27me3_KMS11_plumKD_trgt_rep1.narrowPeak.gz
H3K27me3_KMS11_plumKD_trgt_rep1_1.fq.gz
H3K27me3_KMS11_plumKD_trgt_rep1_2.fq.gz
H3K27me3_KMS11_plumKD_trgt_rep2.bw
H3K27me3_KMS11_plumKD_trgt_rep2.narrowPeak.gz
H3K27me3_KMS11_plumKD_trgt_rep2_1.fq.gz
H3K27me3_KMS11_plumKD_trgt_rep2_2.fq.gz
H3K27me3_KMS11_plumKD_trgt_rep3.bw
H3K27me3_KMS11_plumKD_trgt_rep3.narrowPeak.gz
H3K27me3_KMS11_plumKD_trgt_rep3_1.fq.gz
H3K27me3_KMS11_plumKD_trgt_rep3_2.fq.gz

Genome browser session
(e.g. UCSC)

TBC

Methodology

Replicates

Biological replication, n=3

Sequencing depth	All experiments were sequenced on Illumina NovaSeq X Plus using paired-end sequencing at 150 bp read length.<br>Sequencing depth for each experiment:<br>EZH2_KMS11_endogenous_ctrl_rep1<br>30138204<br>EZH2_KMS11_endogenous_ctrl_rep2<br>40059742<br>EZH2_KMS11_endogenous_ctrl_rep3<br>35598007<br>H3K27me3_KMS11_plumKD_ctrl_rep1<br>37768404<br>H3K27me3_KMS11_plumKD_ctrl_rep2<br>31630025<br>H3K27me3_KMS11_plumKD_ctrl_rep3<br>36915050<br>H3K27me3_KMS11_plumKD_trgt_rep1<br>29186460<br>H3K27me3_KMS11_plumKD_trgt_rep2<br>25135528<br>H3K27me3_KMS11_plumKD_trgt_rep3<br>36837677
Antibodies	H3K27me3 (9733S; CST), HA Ab (901503; Genomax)
Peak calling parameters	ChIP-seq data was processed using the ENCODE ChIP-seq pipeline with the following parameters:<br>bowtie2 -X2000 --mm --threads ${CORES} -x ${BWT2_IDX} -1 ${REPX_1}.fq -2 ${REPX_2}.fq 2 > ${LOG} | samtools view -Su /dev/stdin | samtools sort - > ${REPX}.bam<br>macs2 callpeak -t ${REPX}.tagAlign.gz -c ${INPUT}.tagAlign.gz -f BED -n ${REPX_DIR_PREFIX} -g ${GENOMESIZE} -p 1e-2 --nomodel --shift 0 --extsize ${FRAGLEN} --keep-dup all -B –SPMR
Data quality	Consistency of replicates were ascertained using IDR and PCA.<br>Narrow peaks identified using MACS2 using a pval threshold of 1%<br>EZH2_KMS11_endogenous_ctrl_rep1.narrowPeak.gz<br>147480<br>EZH2_KMS11_endogenous_ctrl_rep2.narrowPeak.gz<br>87981<br>EZH2_KMS11_endogenous_ctrl_rep3.narrowPeak.gz<br>123494<br>H3K27me3_KMS11_plumKD_ctrl_rep1.narrowPeak.gz<br>135009<br>H3K27me3_KMS11_plumKD_ctrl_rep2.narrowPeak.gz<br>158444<br>H3K27me3_KMS11_plumKD_ctrl_rep3.narrowPeak.gz<br>148162<br>H3K27me3_KMS11_plumKD_trgt_rep1.narrowPeak.gz<br>134099<br>H3K27me3_KMS11_plumKD_trgt_rep2.narrowPeak.gz<br>112046<br>H3K27me3_KMS11_plumKD_trgt_rep3.narrowPeak.gz<br>116103
Software	ChIP-seq analyses were performed using bowtie2 for alignments and MACS2 for peak calling.<br>Differential binding and integration was performed in R with DEseq2.

Flow Cytometry

Plots

Confirm that:

☐ The axis labels state the marker and fluorochrome used (e.g. CD4-FITC).

☐ The axis scales are clearly visible. Include numbers along axes only for bottom left plot of group (a 'group' is an analysis of identical markers).

☐ All plots are contour plots with outliers or pseudocolor plots.

☐ A numerical value for number of cells or percentage (with statistics) is provided.

Methodology

Sample preparation	Cell viability assay (AnnexinV): Cells were washed with PBS and re-suspended in Annexin V binding buffer and stained with Alexa Fluor™ 350 Annexin V conjugate for 15min at room temperature in the dark before being analysed by FACs.
Instrument	FACS analysis: BD LSRFortessa™ X-20
Software	BD FACSDiva™ used during FACS<br>Additional analysis done using FlowJo software
Cell population abundance	*Describe the abundance of the relevant cell populations within post-sort fractions, providing details on the purity of the samples and how it was determined.*
Gating strategy	For all samples, FSC/SSC gate was used for the starting cell population followed by doublets exclusion using FSC-W/FSC-A and/or SSC-W/SSC-A before gating for fluorescence markers.<br>Cell viability/AnnexinV staining: Unstained cells were used as negative control to gate for Annexin V negative (live) cells.

☐ Tick this box to confirm that a figure exemplifying the gating strategy is provided in the Supplementary Information.

Magnetic resonance imaging

Experimental design

Design type	*Indicate task or resting state; event-related or block design.*
Design specifications	*Specify the number of blocks, trials or experimental units per session and/or subject, and specify the length of each trial or block (if trials are blocked) and interval between trials.*
Behavioral performance measures	*State number and/or type of variables recorded (e.g. correct button press, response time) and what statistics were used to establish that the subjects were performing the task as expected (e.g. mean, range, and/or standard deviation across subjects).*

Acquisition

Imaging type(s)	*Specify: functional, structural, diffusion, perfusion.*
Field strength	*Specify in Tesla*
Sequence & imaging parameters	*Specify the pulse sequence type (gradient echo, spin echo, etc.), imaging type (EPI, spiral, etc.), field of view, matrix size, slice thickness, orientation and TE/TR/flip angle.*
Area of acquisition	*State whether a whole brain scan was used OR define the area of acquisition, describing how the region was determined.*

Diffusion MRI ☐ Used ☒ Not used

Preprocessing

Preprocessing software	*Provide detail on software version and revision number and on specific parameters (model/functions, brain extraction, segmentation, smoothing kernel size, etc.).*
Normalization	*If data were normalized/standardized, describe the approach(es): specify linear or non-linear and define image types used for transformation OR indicate that data were not normalized and explain rationale for lack of normalization.*
Normalization template	*Describe the template used for normalization/transformation, specifying subject space or group standardized space (e.g. original Talairach, MNI305, ICBM152) OR indicate that the data were not normalized.*
Noise and artifact removal	*Describe your procedure(s) for artifact and structured noise removal, specifying motion parameters, tissue signals and physiological signals (heart rate, respiration).*
Volume censoring	*Define your software and/or method and criteria for volume censoring, and state the extent of such censoring.*

Statistical modeling & inference

Model type and settings	*Specify type (mass univariate, multivariate, RSA, predictive, etc.) and describe essential details of the model at the first and second levels (e.g. fixed, random or mixed effects; drift or auto-correlation).*
Effect(s) tested	*Define precise effect in terms of the task or stimulus conditions instead of psychological concepts and indicate whether ANOVA or factorial designs were used.*

Specify type of analysis: ☐ Whole brain ☐ ROI-based ☐ Both

Statistic type for inference

(See Eklund et al. 2016)

Specify voxel-wise or cluster-wise and report all relevant parameters for cluster-wise methods.

Correction

Describe the type of correction and how it is obtained for multiple comparisons (e.g. FWE, FDR, permutation or Monte Carlo).

Models & analysis

n/a	Involved in the study
☒	☐ Functional and/or effective connectivity
☒	☐ Graph analysis
☒	☐ Multivariate modeling or predictive analysis
